# Sequence Versus Composition: What Prescribes IDP Biophysical Properties?

**DOI:** 10.3390/e21070654

**Published:** 2019-07-03

**Authors:** Jiří Vymětal, Jiří Vondrášek, Klára Hlouchová

**Affiliations:** 1 Institute of Organic Chemistry and Biochemitry of the Czech Academy of Sciences, Flemingovo náměstí 2, 166 10 Praha 6 Prague, Czech Republic; 2 Department of Cell Biology, Faculty of Science, Charles University, Viničná 7, 128 43 Praha 2 Prague, Czech Republic

**Keywords:** IDP, IDR, sequence randomization, secondary structure prediction, aggregation propensity

## Abstract

Intrinsically disordered proteins (IDPs) represent a distinct class of proteins and are distinguished from globular proteins by conformational plasticity, high evolvability and a broad functional repertoire. Some of their properties are reminiscent of early proteins, but their abundance in eukaryotes, functional properties and compositional bias suggest that IDPs appeared at later evolutionary stages. The spectrum of IDP properties and their determinants are still not well defined. This study compares rudimentary physicochemical properties of IDPs and globular proteins using bioinformatic analysis on the level of their native sequences and random sequence permutations, addressing the contributions of composition versus sequence as determinants of the properties. IDPs have, on average, lower predicted secondary structure contents and aggregation propensities and biased amino acid compositions. However, our study shows that IDPs exhibit a broad range of these properties. Induced fold IDPs exhibit very similar compositions and secondary structure/aggregation propensities to globular proteins, and can be distinguished from unfoldable IDPs based on analysis of these sequence properties. While amino acid composition seems to be a major determinant of aggregation and secondary structure propensities, sequence randomization does not result in dramatic changes to these properties, but for both IDPs and globular proteins seems to fine-tune the tradeoff between folding and aggregation.

## 1. Introduction

While intrinsically disordered proteins and regions (IDPs/IDRs) compose a significant part of the proteome, their nature and disorder-function mechanism of activity represent long-missed biochemical paradigms. Their occurrence increases with organism complexity, which is mirrored in the portfolio of IDP/R activities, such as signaling, recognition, and translation/transcription regulation. In addition, IDP/Rs are considered more promiscuous and evolvable than globular proteins [1].

The plethora of IDP/R properties and functions is so broad that the scientific community has been struggling to establish a simple classification strategy. Different classification schemes have been based on function, functional features, sequence motifs or biophysical properties [2]. The simplest structural distinction can be made between IDP/Rs that (i) fold upon binding or as a response to a specific environment, and (ii) sequences that, to our best knowledge, remain unfolded, i.e., “induced fold” and “unfoldable” IDPs (as used throughout this report).

It is generally agreed that all the distinct functional and structural properties of IDP/Rs are rooted in their amino acid sequences [3]. It was noticed soon after the first collection of IDP/Rs that there is a consequential bias in both the amino acid compositions and sequences [3,4]. First, IDP/Rs are depleted in the “order-promoting” residues (CWYFILHVNM) and enriched in “disorder-promoting” residues (KEQSPRGA), bringing together a relatively high net charge with low mean hydrophobicity. Second, the bias is often exacerbated by low complexity regions in IDP/Rs containing multiple repeats [5]. Because of the lack of structural constraints, IDP/Rs are considered less restricted to explore a larger sequence space [5]. This could imply that the biophysical properties of IDPs are easier to maintain than those of globular proteins. To what extent these properties result from the globular/intrinsically disordered protein amino acid composition, and to what extent they are optimized by the sequence of amino acids in the protein chains, is not known.

In this study, we address these issues by analyzing physicochemical properties and performing an amino acid sequence permutation experiment. Rudimentary properties of intrinsically disordered and structured globular protein datasets are compared using sets of bioinformatic analyses on the level of their native sequences and their sequence permutations. While the amino acid composition is preserved during explored permutations, the contributions of composition versus sequence to selected protein properties is directly addressed. Here, we report how protein composition determines the spectrum of the basic biophysical properties that a sequence can adopt. The most general classes of IDPs (induced fold and unfoldable) can be distinguished based on these properties. On the other hand, the spectrum of properties for a fixed composition appears to be quite narrow for aggregation, while sequence rearrangements seem to have a slightly more pronounced impact on fine-tuning secondary structure elements.

## 2. Materials and Methods 

### 2.1. Selection of Protein Datasets

The DisProt dataset was constructed from the DisProt database (DisProt 7 v0.5 11-05-2017, www.disprot.org) [6]. A disordered region from the Disprot database was included in the dataset if its length was in the range of 50 to 150 amino acid residues. This procedure resulted in 361 selected sequences (Table S1). No other selection criteria were applied. Additionally, the induced fold and unfoldable subsets of the DisProt datasets were selected (see below in the Methods, Table S1 and Figure S1).

The PDB dataset was created based on a search performed at the RCSB web interface in February 2019, querying a list of proteins resolved by x-ray crystallography with a monomeric single chain of length between 50 and 150 amino acid residues without modified residues or other molecules, such as nucleic acids or ligands, present in the complex. The search was further limited to representative structures at 30% sequence homology, and resulted in 1043 structures. The content of the preselected structure files was analyzed, and only protein structures without stabilizing cysteine bridges, organic or metallic ligands, and missing residues (tolerating at most 5 residues at the N- and C-terminal of a sequence) were considered suitable representatives of well folded stable proteins. Applying these criteria restricted the final PDB dataset to 259 proteins (Table S1 and Figure S1). 

### 2.2. Bioinformatics Analysis

Prediction of secondary structures was performed using a consensus predictor constructed for this purpose to increase the robustness of the prediction and minimize the bias of any single algorithm. It utilizes outputs of various secondary structure predictors, namely spider3, psipred, predator, jnet, simpa and GOR IV [7,8,9,10,11,12]. Each prediction method employs different algorithms or parameters. Spider3, psipred and jnet are based on neural networks, nevertheless, they use different architecture of the networks (size of the input layer, number and size of hidden layers) and were parametrized on different protein sets. GOR IV, on the other hand, is a bayesian method developed in the framework of information theory. It predicts the secondary structure propensities of a sequence on the basis of observed statistics of individual amino acids in the context of their nearest neighbors. Simpa96 transfers secondary structure propensities from a database of 7-residue fragments by homology. A completely different strategy is adopted by Predator, which assigns secondary structure according to a predicted non-local hydrogen bond network [7,8,9,10,11,12].

The final assignment of secondary structure followed the most frequently predicted secondary structure element at each amino acid position. In the case of ambivalent counts, the impact of individual predictors was prioritized in the listed order. No predictor was allowed to use homology information and build sequence alignments that might prevent high-throughput processing of protein sequences. This consensus method provided 73% accuracy, as measured on the PDB set (see Table S2). 

Protein aggregation was predicted by the ProA algorithm in a protein prediction mode [13].

The disorder index was calculated as an average of MobiDB-lite scores over a particular sequence region [14]. The MobiDB-lite predictor is consensus based, optimized for high specificity (against false positives), and trained to recognize long sequence disorder. MobiDB-lite scores for the DisProt dataset were adopted from the MobiDB 3.0 database (http://mobidb.bio.unipd.it/) [6]. 

### 2.3. Survey of PDB-DisProt Overlap

The sequences of the DisProt dataset were investigated for their occurrence in the PDB (February 2019). Each sequence from the DisProt dataset were exhaustively compared with all protein sequences of all PDB entries. If a sequence overlap longer than 20 amino acid residues was found, the match was further examined. The hit was accepted if the sequence overlap included the whole DisProt region or the whole PDB chain. Additionally, the hit was also accepted if it lacked complete overlap of one or the other sequence while no sequence conflict was detected. This occurred most often when a PDB chain started or ended within a DisProt region. The exact match at all positions in the overlap was required, but this requirement was alleviated for the first and last three amino acids of a PDB chain, which possibly differ from the native sequence.

All hits were visually reviewed, and structures with a complex fold were included into an “induced fold” subset of the DisProt dataset (Figures S1 and S2). The condition of the complex fold requires the existence of a secondary structure network and the presence of a non-negligible amount of tertiary interactions, preferably forming a hydrophobic core. Single secondary structure elements, such as a helices and isolated pre-structured motifs, were not considered. Applying these criteria, the 30 best-matching sequences were finally selected (Figures S1 and S2). 

As opposed to the induced subset, the “unfoldable” subset was constructed. It was comprised of the 30 sequences of the DisProt dataset with the highest average MobiDB-lite scores and no 3D structure recorded in PDB. These sequences are supposed to be highly disordered and unable to fold into structures similar to globular proteins (Figure S1).

### 2.4. Sequence Permutations

Permutations of protein sequences were generated randomly for all amino acid residues at all positions at the same time. No correlations between individual positions were constrained or reintroduced. Therefore, this procedure produced totally random protein sequences of the same defined composition. Permutations were generated using a *shuffle* method of the standard *random* python module. 

One thousand different random permutations were produced for each protein sequence in both datasets. All of these artificial sequences were examined by our bioinformatics analysis for secondary structure content and aggregation propensity. 

### 2.5. Statistical Analysis

Analysis and data processing were conducted by in-house developed python scripts using matplotlib for plotting [15]. Principal component analyses were performed using the scikit-learn machine learning package on the combined DisProt and PDB datasets [16].

## 3. Results

### 3.1. Secondary Structure and Aggregation Propensity Can Distinguish Between Induced Fold and Unfoldable IDPs

**Figure 1:**
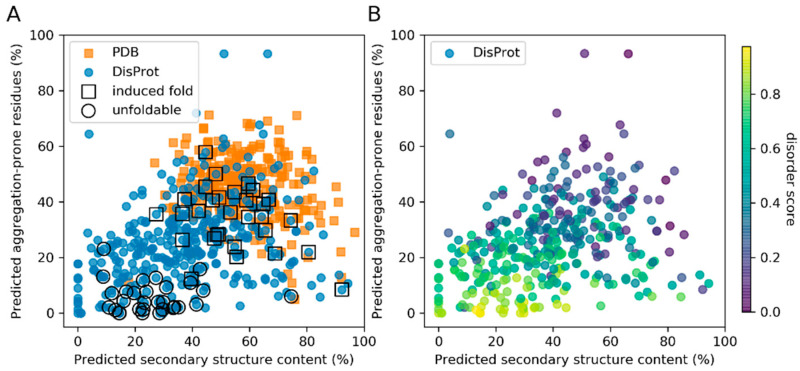
Scatter plot showing predicted secondary structure content (x-axis) and aggregation propensity (y-axis) of the PDB (blue) and DisProt (orange) datasets. The Disprot dataset is further annotated for classified examples of induced fold (by squares) and unfoldable (by circles) intrinsically disordered proteins (IDPs) in (**A**), which belong also to the most disordered Disprot predicted proteins by MobiDB-lite score in (**B**).

Two datasets were used in this study: (i) Folded stable proteins extracted from the PDB including 259 unique sequences, and (ii) a DisProt dataset of 361 sequences selected from the DisProt database. Both were limited to a size of 50–150 amino acid long sequences (see the Methods section for more details). Consensus secondary structure and disorder predictions of these datasets were plotted against predicted features important to protein aggregation using the ProA algorithm (Figure 1) [13]. The consensus secondary structure prediction is based on the performance of six predictors (selected to be able to perform in a high-throughput regime and to be based on different algorithms), which have been tested on the PDB dataset of this study, reporting a 73% average precision (Figure S3 and Table S2). We assume that the consensus-based approach provides higher robustness in predictions on disordered and permuted protein sequences than individual predictors, while maintaining high precision for regularly folded proteins.

Albeit there are exceptions, there is a general trend of increased aggregation propensity with increased secondary structure content (Figure 1A). Although IDPs have generally lower aggregation propensity, they represent a class of proteins with a very broad spectrum of physico-chemical properties. IDPs with low predicted disorder content cluster at a very similar secondary structure/aggregation part of the spectrum as the PDB dataset (Figure 1B). Many of such IDPs belong to a subclass of IDPs that fold upon an external trigger (i.e., “induced fold IDPs”), distinguishing them from IDPs that remain unfoldable based on the available knowledge (Figure 1A,B and Figure S1). The induced fold subset contains prominent examples of proteins reported as disordered that gain a complex fold upon binding an interaction partner or a cofactor (Figure S2). On the other hand, the unfoldable subset represents the most likely disordered regions predicted by the MobiDB-lite predictor (Figure S1) (see the Methods section for more details).

### 3.2. The Composition of IDPs Partly Overlaps with Narrower Globular Protein Composition

**Figure 2:**
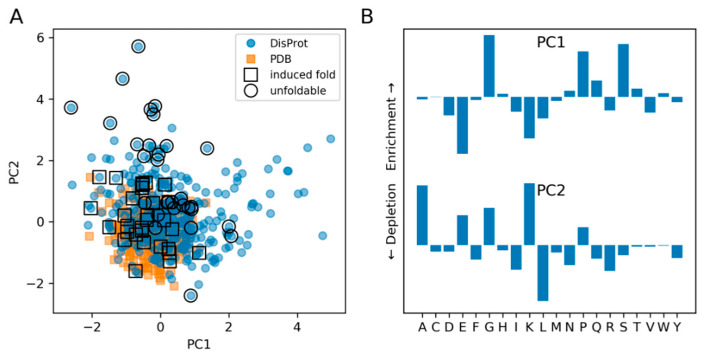
Compositional distribution of the PDB and DisProt datasets. Projections to the principal components (PC1 and PC2) are presented in (**A**), the Disprot dataset has the induced fold (squares) and unfoldable (circles) representatives highlighted. Contributions of individual amino acids to PC1 and PC2 are visualized in (**B**).

Besides the similarity in secondary structures and aggregation propensities, the induced fold DisProt proteins also colocalize with PDB proteins in their compositional analysis by Principal Component Analysis (PCA) (Figure 2A). PCA performed on normalized amino acid frequencies in each protein sequence (20-dimensional vectors) revealed that the overall distribution is broader for the DisProt dataset relative to the PDB proteins. PCA brought another distinguishable characteristic. The unfoldable DisProt proteins appear to be enriched in the PC2 of the two principal components. The amino-acid composition of sequences contributing to PC1 is enriched in glycine, proline, serine and glutamine, but it is depleted in glutamate and lysine. On the other hand, a high content of glutamate, lysine and alanine defines the PC2 (Figure 2B). 

### 3.3. Sequence Permutation Experiments Suggest a Dominant Role of Composition 

**Figure 3:**
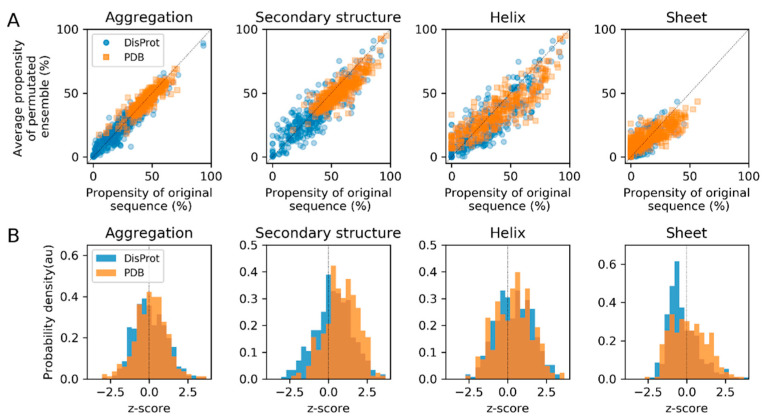
Analysis of properties of the DisProt and PDB datasets, showing the (**A**) mean values and (**B**) z-score of aggregation and secondary structure propensity after 1000 sequence permutations, compared to the original sequence.

Both datasets were completely permuted 1000 times on the sequence level (conserving the composition of individual proteins) and compared with the original sequence to identify properties that have been significantly affected by evolution on the level of amino acid sequence. No significant effects on aggregation and secondary structure propensities were observed using the predictors after sequence permutations, i.e., there was notable correlation of the properties of original and permuted sequences (Pearson’s *r* in the range of 0.73–0.94 for all datasets properties) (Figure 3). This suggests that the role of composition (versus particular sequences) on these properties is dominant. Protein composition generally determines the spectrum of properties that a specific sequence can occupy, and this spectrum is especially narrow for aggregation propensity (Figure 3A). A minor role of the sequence is observed in favor of secondary structure formation of PDB proteins (Figure 3B, secondary structure) and against beta-sheet formation of DisProt proteins (Figure 3B, sheet), as deduced from the z-score related to the original sequence in the distribution of its permuted variants.

## 4. Discussion

The goal of this study is to quantify the importance of the amino acid composition versus sequence on the physicochemical properties of IDPs and globular proteins. As expected, IDPs have, on average, lower predicted secondary structure content, lower aggregation propensity and biased amino acid composition. This is in accordance with earlier studies [4,17,18,19]. However, IDPs exhibit a broad range of these properties. We can conclude that IDPs that are foldable upon external triggering (induced fold) have similar compositions and secondary structure/aggregation propensities as folded proteins, distinguishing them from unfoldable IDPs. Amino acid composition seems to be the major determinant of this distinction. Unfoldable IDPs are, on average, more enriched, e.g., in glutamate and lysine (located on PC2-axis in Figure 2). Some other representatives of IDPs show a high content of glycine, proline and serine (on PC1-axis in Figure 2). These findings recapitulate the fact that there are different flavors of disorder, which are distinguishable by amino acid composition and biological function [20]. 

Previous studies using polymer theory and biophysical measurements have defined three distinct compositional classes based on the fraction of charged versus polar residues [21]. One of these classes (polar tracts) was reported to be enriched in polar amino acids, which probably corresponds to the PC1 component of our analysis (Figure 2). In further agreement, the PC2 component of our analysis (enriched in charged residues) corresponds to the classes of polyampholytes/polyelectrolytes that would have an intrinsic tendency to populate expanded conformations [22]. Our study is therefore in accordance with the IDP compositional bias reported previously. In addition, we show that compositional analysis and secondary structure prediction can further differentiate the induced fold IDP class that is very similar to globular proteins in these characteristics, and would not fit easily into categories described by polymer theory.

The sequence permutation experiment was used to resolve the contributions of sequence versus composition to globular and IDPs. Upon sequence randomization within a given composition, IDP sequences increased in beta-sheet content, unlike folded proteins that decreased moderately in secondary structure content. Sequence rearrangements appeared to have a smaller impact on the aggregation propensity. However, it has been recognized that there is a tradeoff between folding and aggregation propensity, and they cannot be optimized simultaneously [23]. While highly aggregating sequence stretches are prevented, some extent of aggregation propensity is tolerated, especially at the cost of achieving protein structure [24]. Loss of secondary structure content upon sequence randomization of folded proteins is suggestive of this tradeoff, where aggregation propensity can be counteracted by folding. Similarly, suppression of beta-sheet content in IDPs could minimize their risk of aggregation. This is in agreement with a previous study reporting that IDP compaction is preferentially achieved by forming alpha-helices [17]. Interestingly, randomized IDP and PDB proteins have been reported to have an increased propensity for amyloidogenesis [17]. For a given composition, protein sequence can probably be an important variable of structural compaction [25]. 

We can conclude that changes in properties after sequence randomization are not dramatic, as observed in Figure 3. This is in agreement with the observation that the secondary structure and aggregation propensity of random sequences are overall similar to biological proteins, and that even de novo genes have aggregation propensities similar to existing proteins [26,27]. Moreover, our results are not surprising, as compositions have been reported to be conserved among orthologs of IDPs, even if their sequences are poorly conserved, and IDP composition has been suggested to define an IDP functional classification scheme based on these finding [22,28]. 

However, it is important to keep in mind that the consensus secondary structure and aggregation predictions used in this study both have an accuracy of 70–80% (Table S2) [13]. It is possible that some of the observed phenomena were biased by the predictions. 

Overall, our study suggests that amino acid composition is the most important parameter of behavior within the IDP class. Sequence rearrangements have limited effects on the physicochemical properties, both on aggregation as well as secondary structure content, based on bioinformatic predictions. Nevertheless, further experimental verification of this outcome is demanded.

## Supplementary Materials

The following are available online at https://www.mdpi.com/1099-4300/21/7/654/s1: Figure S1. Available PDB structures of the DisProt dataset and schematic representation of unfoldable and induced fold IDP selection criteria, Figure S2. 3D Structures of the induced fold DisProt subset, Figure S3. Bioinformatic prediction vs. secondary structure assignment from experimental data of the PDB dataset, Table S1. List of protein sequences with DisProt/PDB IDs (the Disprot dataset has the induced fold IDPs highlighted in red and the unfoldable IDPs are in blue), Table S2. Justification of secondary structure predictors measured on PDB dataset (the reported numbers represent fraction of correctly predicted residues in a three state model - helix, sheet, coil).
