# Supplementary material for: Sequence Versus Composition: What Prescribes IDP Biophysical Properties?
Source: Entropy (Basel). 2019 Jul 3;21(7):654. doi: 10.3390/e21070654 (PMC7515148; doi:10.3390/e21070654)
Supplement: Supplementary file 1 [file entropy-21-00654-s001.zip › SuplMaterial/Vymetal_TableS1.pdf]

Table S1: List of protein sequences with DisProt/PDB IDs
(the Disprot dataset has the induced fold IDPs highlighted in red and the unfoldable IDPs are in blue)

**PDB dataset**		
**#**	**PDB ID**	**sequence**
1	1A32	LTQERKREIIEQFKVHENDTGSPEVQIAILTEQINNLNEHLRVHKKDHHSRRGLLKMVGKRRRLLAYLRNKDVARYREIVEKLGL
2	1BKR	KSAKDALLLWCQMKTAGYPNVNIHNFTTSWRDGMAFNALIHKHRPDLIDFDKLKKSNAHYNLQNAFNLAEQHLGLTKLLDPEDISVDHPDEKSIITYVVTYYHYFSKM
3	1BM8	QIYSARYSGVDVYEFIHSTGSIMKRKKDDWVNATHILKAANFAKAKRTRILEKEVLKETHEKVQGGFGKYQGTWVPLNIAKQLAEKFSVYDQLKPLFDF
4	1BZ4	SGQRWELALGRFWDYLRWVQTLSEQVQEELLSSQVTQELRALMDETMKELKAYKSELEEQLTPVAEETRARLSKELQAAQARLGADMEDVCGRLVQYRGEVQAMLGQSTEELRVRLASHLRKLRKRLLRDADDLQKRLAVYQAG
5	1CDZ	ELPDFFQGKHFFLYGEFPGDERRKLIRYVTAFNGELEDYMSDRVQFVITAQEWDPSFEEALMDNPSLAFVRPRWIYSCNEKQKLLPHQLYGVVPQA
6	1CQY	TPVMQTIVVKNVPTTIGDTVYITGNRAELGSWDTKQYPIQLYYDSHSNDWRGNVVLPAERNIEFKAFIKSKDGTVKSWQTIQQSWNPVPLKTTSHTSSW
7	1DSL	TFRMRIYERDDFRGQMSEITDDCPSLQDRFHLTEVHSLNVLEGSWVLYEMPSYRGRQYLLRPGEYRRYLDWGAMNAKVGSLRRVMDFY
8	1DT4	MKDVVEIAVPENLVGAILGKGGKTLVEYQELTGCRIQISKKGEFLPGTRNRKVTITGTPAATQAAQYLITQRI
9	1DXS	SLVSFLTGLGCPNCIEYFTSQGLQSIYHLQNLTIEDLGALKIPEQYRMTIWRGLQDL
10	1EAN	GELVRTDSPNFLSSVLPTHWRSNKTLPIAFKVVALGDVPDGTLVTVMAGNDENYSAELRNATAAMKNQVARFNDLRFVGRSGRGKSFTLTITVFTNPPQVATYHRAIKITVDGP
11	1EOE	ERVVINVSGLRFETQLKTLNQFPDTLLGNPQKRNRYYDPLRNEYFFDRNRPSFDAILYFYQSGGRLRRPRNVPLDVFSEEIKFYELGENAFERYREDEGF
12	1EW4	MNDSEFHRLADQLWLTIEERLDDWDGDSDIDCEINGGVLTITFENGSKIIINRQEPLHQVWLATKQGGYHFDLKGDEWICDRSGETFWDLLEQAATQQAGETVSFR
13	1EYH	HNYSEAEIKVREATSNDPWGPSSSLMSEIADLTYNVVAFSEIMSMIWKRLNDHGKNWRHVYKAMTLMEYLIKTGSERVSQQCKENMYAVQTLKDFQYVDRDGKDQGVNVREKAKQLVALLRDEDRLREERAHALKTKEKLAQTA
14	1F0M	YTSFNTVDEWLEAIKMGQYKESFANAGFTSFDVVSQMMMEDILRVGVTLAGHQKKILNSIQVMRAQMNQIQ
15	1GV2	ELIKGPWTKEEDQRVIKLVQKYGPKRWSVIAKHLKGRIGKQCRERWHNHLNPEVKKTSWTEEEDRIIYQAHKRLGNRWAEIAKLLPGRTDNAIKNHWNSTMR
16	1GYU	MIPSITAYSKNGLKIEFTFERSNTNPSVTVITIQASNSTELDMTDFVFQAAVPKTFQLQLLSPSSSVVPAFNTGTITQVIKVLNPQKQQLRMRIKLTYNHKGSAMQDLAEVNNFPPQSWQ
17	1H3Q	MSGSFYFVIVGHHDNPVFEMEFLPPGKAESKDDHRHLNQFIAHAALDLVDENMWLSNNMYLKTVDKFNEWFVSAFVTAGHMRFIMLHDVRQEDGIKNFFTDVYDLYIKFAMNPFYEPNSPIRSSAFDRKVQFLGKKHLLS
18	1HUF	LSLSDLHRQVSRLVQQESGDCTGKLRGNVAANKETTFQGLTIASGARESEKVFAQTVLSHVANVVLTQEDTAKLLQSTVKHNLNNYDLRSVGNGNSVLVSLRSDQMTLQDAKVLLEAALRQES
19	1I2T	HRQALGERLYPRVQAMQPAFASKITGMLLELSPAQLLLLLASEDSLRARVDEAMELIIAHG
20	1J27	MKAYLGLYTARLETPARSLKEKRALIKPALERLKARFPVSAARLYGLDAWGYEVVGFTLLGNDPAWVEETMRAAARFLAEAGGFQVALEEFRLEAFEL
21	1J74	VKVPRNFRLLEELEEGQKGVGDGTVSWGLEDDEDMTLTRWTGMIIGPPRTNYENRIYSLKVECGPKYPEAPPSVRFVTKINMNGINNSSGMVDARSIPVLAKWQNSYSIKVVLQELRRLMMSKENMKLPQPPEGQTYNN
22	1JB3	ELQRREEEANVVLTGTVEEIMNVDPVHHTYSCKVRVWRYLKGKDIVTHEILLDGGNKVVIGGFGDPLICDNQVSTGDTRIFFVNPAPQYMWPAHRNELMLNSSLMRITLRNLEEVEHCVEEHRKLLA
23	1JMW	MNQQGFVISNELRQQQSELTSTWDLMLQTRINLARSAARMMMDASNQQSSAKTDLLQNAKTTLAQAAAHYANFKNMTPLPAMAEASANVDEKYQRYQAALAELIQFLDNGNMDAYFAQPTQGMQNALGEALGNYARVSENLYRQTF
24	1JOS	RSDRVAQEIQKEIAVILQREVKDPRIGMVTVSDVEVSSDLSYAKIFVTFLFDHDEMAIEQGMKGLEKASPYIRSLLGKAMRLRIVPEIRFIYDQSLVEGM
25	1K40	NDKVYENVTGLVKAVIEMSSKIQPAPPEEYVPMVKEVGLALRTLLATVDETIPALPASTHREIEMAQKLLNSDLGELISKMKLAQQYVMTSLQQEYKKQMLTAAHALAVDAKNLLDVIDQARLKML
26	1K6K	MLNQELELSLNMAFARAREHRHEFMTVEHLLLALLSNPSAREALEACSVDLVALRQELEAFIEQTTPVLPASEEERDTQPTLSFQRVLQRAVFHVQSSGRNEVTGANVLVAIFSEQESQAAYLLRKHEVSRLDVVNFISHGT
27	1KU3	ELEKALSKLSEREAMVLKMRKGLIDGREHTLEEVGAYFGVTRERIRQIENKALRKLKYHES
28	1L2P	TDQLKKAKAEAQVIIEQANKRRSQILDEAKAEAEQERTKIVAQAQAEIEAERKRAREELRK
29	1LMI	SAYPITGKLGSELTMTDTVGQVVLGWKVSDLKSSTAVIPGYPVAGQVWEATATVNAIRGSVTPAVSQFNARTADGINYRVLWQAAGPDTISGATIPQGEQSTGKIYFDVTGPSPTIVAMNNGMEDLLIWEP
30	1LN4	MDLSTKQKQHLKGLAHPLKPVVLLGSNGLTEGVLAEIEQALEHHELIKVKIATEDRETKTLIVEAIVRETGACNVQVIGKTLVLYRPTKERKISLPLE

31	1LOU	MRRYEVNIVLNPNLDQSQLALEKEIIQRAAENYGARVEKVEELGLRRLAYPIAKDPQGYFLWYQVEMPEDRVNDLARELRIRDNVRRVMVVKSQEPF
32	1LU4	ADERLQFTATTLSGAPFDGASLQGKPAVLWFWTPWCPFCNAEAPSLSQVAAANPAVTFVGIATRADVGAMQSFVSKYNLNFTNLNDADGVIWARYNVPWQPAFVFYRADGTSTFVNNPTAAMSQDELSGRVAAL
33	1MB1	NQIYSARYSGVDVYEFIHSTGSIMKRKKDDWVNATHILKAANFAKAKRTRILEKEVLKETHEKVQGGFGKYQGTWVPLNIAKQLAEKFSVYDQLKPLF
34	1MHN	LQQWKVGDKCSAIWSEDGCIYPATIASIDFKRETCVVVYTGYGNREEQNLSDLLSPICE
35	1MJC	SGKMTGIVKWFNADKGFGFITPDDGSKDVFVHFSAIQNDGYKSLDEGQKVSFTIESGAKGPAAGNVTSL
36	1NG6	MSLLERLNQDMKLYMKNREKDKLTVVRMVKASLQNEAIKLKKDSLTEDEELTVLSRELKQRKDSLQEFSNANRLDLVDKVQKELDILEVYLPEQLSEEELRTIVNETIAEVGASSKADMGKVMGAIMPKVKGKADGSLINKLVSSQLS
37	1O3X	AANKLIKEMVQEDQKRMEKISKRVNAIEEVNNNVKLLTEMVMSHSQGGAAAGSSEDLMKELYQRCERMRPTLFRLASDTEDNDEALAEILQANDNLTQVINLYKQLVRGEEV
38	1O8X	GLDKYLPGIEKLRRGDGEVEVKSLAGKLVFFYFSASWCPPARGFTPQLIEFYDKFHESKNFEVVFCTWDEEEDGFAGYFAKMPWLAVPFAQSEAVQKLSKHFNVESIPTLIGVDADSGDVVTTRARATLVKDPEGEQFPWKDA
39	1OZ9	KNRVLVKLKKRKVRKDKIEKWAELALSALGLNNVELSVYITDDQEIRELNKTYRKKDKPTDVLSFPMGEEFGGYKILGDVVISQDTAERQARELGHSLEEEVKRLIVHGIVHLLGYDHEKGGEEEKKFRELENYVLSKLSK
40	1P4P	SKKLTRSNGTTLEYSQITDADNATKAVETLKNSIKLEGSLVVGKTTVEIKEGTVTLKREIEKDGKVKVFLNDTAGSNKKTGKWEDSTSTLTISADSKKTKDLVFLTDGTITVQQYNTAGTSLEGSASEIKNLSELKNALK
41	1PGX	ELTPAVTTYKLVINGKTLKGETTTKAVDAETAEKAFKQYANDNGVDGVWTYDDATKTFTVTEMVTEVPVA
42	1Q2Y	MKAVIAKNEEQLKDAFYVREEVFVKEQNVPAEEEIDELENESEHIVVYDGEKPVGAGRWRMKDGYGKLERICVLKSHRSAGVGGIIMKALEKAAADGGASGFILNAQTQAVPFYKKHGYRVLSEKEFLDAGIPHLQMMKD
43	1QAU	NVISVRLFKRKVGGLGFLVKERVSKPPVIISDLIRGGAAEQSGLIQAGDIILAVNDRPLVDLSYDSALEVLRGIASETHVVLILRGPEGFTTHLETTFTGDGTPKTIRVTQP
44	1QZM	SGYTEDEKLNIAKRHLLPKQIERNALKKGELTVDDSAIIGIIRYYTREAGVRGLEREISKLCRKAVKQLLLDKSLKHIEINGDNLHDYLGVQRF
45	1R69	SISSRVKSKRIQLGLNQAELAQKVGTTQQSIEQLENGKTKRPRFLPELASALGVSVDWLLNGT
46	1R6J	GAMDPRTITMHKDSTGHVGFIFKNGKITSIVKDSSAARNGLLTEHNICEINGQNVIGLKDSQIADILSTSGTVVTITIMPAF
47	1R9H	KIDITPKKDGGVLKLIKKEGQGVVKPTTGTTVKVHYVGTLENGTKFDSSRDRGDQFSFNLGRGNVIKGWDLGVATMTKGEVAEFTIRSDYGYGDAGSPPKIPGGATLIFEVELFEWSA
48	1SRV	GYQFDKGYISPYFVTNPETMEAVLEDAFILIVEKKVSNVRELLPILEQVAQTGKPLLIIAEDVEGEALATLVVNKLRGTLSVAAVKAPGFGDRRKEMLKDIAAVTGGTVISEELGFKLENATLSMLGRAERVRITKDETTIVGGK
49	1T3Y	ATKIDKEACRAAYNLVRDDGSAVIWVTFKYDGSTIVPGEQGAEYQHFIQQCTDDVRLFAFVRFTTGDAMSKRSKFALITWIGENVSGLQRAKTGTDKTLVKEVVQNFAKEFVISDRKELEEDFIKSELKKA
50	1TG0	EPEVPFKVVAQFPYKSDYEDDLNFEKDQEIIVTSVEDAEWYFGEYQDSNGDVIEGIFPKSFVAVQG
51	1TIG	INVKEVRLSPTIEEHDFNTKLRNARKFLEKGDKVKATIRFKGRAITHKEIGQRVLDRLSEACADIAVVETAPKMDGRNMFLVLAPKND
52	1TP6	CAYRREIHHAHVAIRDWLAGDSRADALDALMARFAEDFSMVTPHGVVLDKTALGELFRSKGGTRPGLRIEIDGESLLASGVDGATLAYREIQSDAAGRSERLSTVVLHRDDEGRLYWRHLQETFCG
53	1TQG	GSHMEYLGVFVDETKEYLQNLNDTLLELEKNPEDMELINEAFRALHTLKGMAGTMGFSSMAKLCHTLENILDKARNSEIKITSDLLDKIFAGVDMITRMVDKIVS
54	1TSF	QGVELIARDWIGLMVEVVESPNHSEVGIKGEVVDETQNTLKIMTEKGLKVVAKRGRTFRVWYKGKIMRIKGDLINFRPEDR
55	1TUC	MGPREVTMKKGDILTLLNSTNKDWWKVEVNDRQGFVPAAYVKKLDSGTGKELVLALYDYQE
56	1TZV	MKTPRRRMRLAVFKALFQHEFRRDEDLEQILEEILDETYDKKAKEDARRYIRGIKENLSMIDDLISRYLEKWSLNRLSVVDRNVLRLATYELLFEKDIPIEVTIDEAIEIAKRYGTENSGKFVNGILDRIAKEHAPKEKFE
57	1UCS	NKASVVANQLIPINTALTLIMMKAEVVTPMGIPAEEIPKLVGMQVNRAVPLGTTLMPDMVKNYE
58	1VCC	MRALFYKDGKLFTDNNFLNPVSDDNPAYEVLQHVKIPTHLTDVVVYEQTWEEALTRLIFVGSDSKGRRQYFYGKMHV
59	1WKA	GKLYTLRYEVEGGGFIEIATVRPETVFADQAIAVHPEDERYRHLLGKRARIPLTEVWIPILADPAVEKDFGTGALKVTPAHDPLDYEIGERHGLKPVSVINLEGRMEGERVPEALRGLDRFEARRKAVELFREAGHLVKEEDY
60	1WPA	WIREYPPITSDQQRQLYKRNFDTGLQEYKSLQSELDEINKELSRLDKELDDYREESEEYMAAADEYNRLKQVKGSADYKSKKNHCKQLKSKLSHIKKMVGDYDRQKT
61	1WVN	PLGSQTTHELTIPNNLIGCIIGRQGANINEIRQMSGAQIKIANPVEGSSGRQVTITGSAASISLAQYLINARLS
62	1X6J	MDINNKARIHWACRRGMRELDISIMPFFEHEYDSLSDDEKRIFIRLLECDDPDLFNWLMNHGKPADAELEMMVRLIQTRNRERGPVAI
63	1XAW	WIREYPPITSDQQRQLYKRNFDTGLQEYKSLQSVLDEINKELSRLDKELDDYREESEEYMAAADEYNRLKQVKGSADYKSKKNHCKQLKSKLSHIKKMVGDYDRQKT
64	1Y2F	RKEAVIIMNVAAHHGSELNGELLLNSIQQAGFIFGDMNIYHRHLSPDGSGPALFSLANMVKPGTFDPEMKDFTTPGVTIFMQVPSYGDELQNFKLMLQSAQHIADEVGGVVLDDQRRMMTPQKLREYQDIIREVKDANA
65	1YU5	PTKLETFPLDVLVNTAAEDLPRGVDPSRKENHLSDEDFKAVFGMTRSAFANLPLWKQQNLKKEKGLF

66	1YZM	GSPLLQQIHNITSFIRQAKAAGRMDEVRTLQENLRQLQDEYDQQQT
67	1ZHV	APRIKLKILNGSYGIARLSASEAIPAWADGGGFVSITRTDDELSIVCLIDRIPQDVRVDPGWSCFKFQGPFAFDETGIVLSVISPLSTNGIGIFVVSTFDGDHLLVRSNDLEKTADLLANAGHSLLLEHHHHHH
68	1ZZK	MGPIITTQVTIPKDLAGSIIGKGGQRIKQIRHESGASIKIDEPLEGSEDRIITITGTQDQIQNAQYLLQNSVKQYSGKFF
69	2A0J	SLIGEILPLSHIVLDMEVGSKKRLFEEAGLLLERESSLSHADVFECLFAREKLGSTGLGQGVAIPHGRHAGVKQATGAFIRTREPVGFDAPDGKPVSLIFILLVPENATGEHLEVLSKLAGKFSQKSIRESLMTVSSAEEVRAILT
70	2B8I	IRPYMKALIYETLVNLANQDPEQHATIRQNLYEQLDLPFDKQLALYAGALGPASSGKLENHEAISNAVDSVVQLLEI
71	2BK8	GAMVSGQIMHAVGEEGGHVKYVCKIENYDQSTQVTWYFGVRQLENSEKYEITYEDGVAILYVKDITKLDDGTYRCKVVNDYGEDSSYAELFVKGVRE
72	2BZX	PVFAKAIQKRVPCAYDKTALALEVGDIVKVTRMNINGQWEGEVNGRKGLFPFTHVKIFDP
73	2CGQ	AMEEAINATIQRILRTDRGITANQVLVDDLGFDSLKLFQLITELEDEFDIAISFRDAQNIKTVGDVYTSVAVWF
74	2CIU	SGDTQLFNRAVSMVEKNKDIRSLLQCDDGITGKERLKAYGELITNDKWTRNRPIVSTKKLDKEGRTHHYMRFHVESKKKIALVHLEAKESKQNYQPDFINMYVDVPGEKRYYLIKPKLHPVSN
75	2CJJ	GRPWSAKENKAFERALAVYDKDTPDRWANVARAVEGRTPEEVKKHYEILVEDIKYIESGKVPF
76	2CKX	RPFSVAEVEALVEAVEHLGTGRWRDVKMRAFDNADHRTYVDLKDKWKTLVHTASIAPQQRRGEPVPQDLLDRVLAAHAYWSQQ
77	2CWP	MELYDVDEFWKFQMKVGLVKKAEKIKRTKKLIKLIVDFGNEERTIVTGIADQIPPEELEGKKFIFVVNLKPKKFSGVESQGMLILAETEDGKVYLIPVPEEVPVGARVW
78	2CXC	ITLEELRYISVFHSITGVTAYRCIVDEENNRLIFLVSEGEAGRAIGRGGRLIKLLREALGKNIEVVEYSSDLERIVKNLFPGVKIESINVRERNGVKQVVIKVSEDDKGAAIGKGGKNVKRARLVLSKLFGVEKVVIR
79	2D59	TRPIDGLTDEDIREILTRYKKIALVGASPKPERDANIVMKYLLEHGYDVYPVNPKYEEVLGRKCYPSVLDIPDKIEVVDLFVKPKLTMEYVEQAIKKGAKVVWFQYNTYNREASKKADEAGLIIVANRCMMREHERLLGEK
80	2E1F	QPVISAQEQETQIVLYGKLVEARQKHANKMDVPPAILATNKILVDMAKMRPTTVENVKRIDGVSEGKAAMLAPLLEVIKHFCQTNSVQTDLFSS
81	2EA9	MSNTTWGLQRDITPRLGARLVQEGNQLHYLADRASITGKFSDAECPKLDVVFPHFISQIESMLTTGELNPRHAQCVTLYHNGFTCEADTLGSCGYVYIAVYPT
82	2EIF	MVIIMPGTKQVNVGSLKVGQYVMIDGVPCEIVDISVSKPGKHGGAKARVVGIGIFEKVKKEFVAPTSSKVEVPIIDRRKGQVLAIMGDMVQIMDLQTYETLELPIPEGIEGLEPGGEVEYIEAVGQYKITRVI
83	2END	TRINLTLVSELADQHLMAEYRELPRVFGAVRKHVANGKRVRDFKISPTFILGAGHVTFFYDKLEFLRKRQIELIAECLKRGFNIKDTTVQDISDIPQEFRGDYIPHEASIAISQARLDEKIAQRPTWYKYYGKAIYA
84	2F15	QARPTVIRWSEGGKEVFISGSFNNWSTKIPLIKSHNDFVAILDLPEGEHQYKFFVDGQWVHDPSEPVVTSQLGTINNLIHVKKSDFEVF
85	2FI0	VVMDNIIDVSIPVAEVVDKHPEVLEILVELGFKPLANPLMRNTVGRKVSLKQGSKLAGTPMDKIVRTLEANGYEVIGLD
86	2FI9	HFPGRAPIDAYGNGGFRFADMSHRGSIICIPSGIYGIDMTGPVPTQEDISRVLEESDQIEVLLIGTGVELLRLPEELRVLLWEKRISSDTMSTGAAVRTFNVLLAEDRAVAALLFAVE
87	2FO3	YRIQKELHNFLNNPPINCTLDVHPNNIRIWIVKYVGLENTIYANEVYKLKIIFPDDYPLKPPIVYFLQKPPKHTHVYSNGDICLSLLGDDYNPSLSISGLVLSIISMLS
88	2FQ3	SKWFNLEKIHSIEVQSLPEFFTNRIPSKTPEVYMRYRNFMVNSYRLNPNEYFSVTTARRNVSGDAAALFRLHKFLTKWGLINYQV
89	2FZP	SSGLVPRGSTIEYNEILEWVNSLQPARVTRWGGMISTPDAVLQAVIKRSLVESGCPASIVNELIENAHERSWPQGLATLETRQMNRRYYENYVAKRIPGKQAVVVMACENQHMGDDMVQEPGLVMIFAHGVEEI
90	2GKG	KKILIVESDTALSATLRSALEGRGFTVDETTDGKGSVEQIRRDRPDLVVLAVDLSAGQNGYLICGKLKKDDDLKNVPIVIIGNPDGFAQHRKLKAHADEYVAKPVDADQLVERAGALIGFPE
91	2H36	KQDLEKIESDIINDWTEADDLDDALDFLFMEKVSEFKIKFKDPLKVTEEEYRELLGNYDSSNSVSSNGITIDQYTYDEDDDIMYKLEFTYRKEDNKIYIYEVQGWREK
92	2HAZ	SHMDTPSSPSIDQVEPYSSTAQVQFDEPEATGGVPILKYKAEWRAVGEEVWHSKWYDAKEASMEGIVTIVGLKPETTYAVRLAALNGKGLGEISAASEFKTQPV
93	2HC8	EAIKKLVGLQAKTAVVIRDGKEIAVPVEEVAVGDIVIVRPGEKIPVDGVVVEGESYVDESMISGEPVPVLKSKGDEVFGATINNTGVLKIRATRVGGETLLAQIVKLVEDAMG
94	2HDZ	LPESPKRAEEIWQQSVIGDYLARFKNDRVKALKAMEMTWNNMEKKEKLMWIKKAAEDQKRYERELS
95	2I6V	QEIFQYVRLSQVKRDDKVLGYRVSPGKDPVLFESIGLQDGDMAVALNGLDLTDPNVMNTLFQSMNEMTEMSLTVERDGQQHDVYIQF
96	2IGD	MTPAVTTYKLVINGKTLKGETTTKAVDAETAEKAFKQYANDNGVDGVWTYDDATKTFTVTE
97	2J49	PENYIRAYSMLKNWVDSSLEIYKPELSYIMYPIFIYLFLNLVAKNPVYARRFFDRFSPDFKDFHGSEINRLFSVNSIDHIKENEVASAFQSHKYRITMSKTTLNLLLYFLNENESIGGSLIISVINQHLDPNIV
98	2J9V	HHHMFNAKYVAEATGNFITVMDALKLNYNAKDQLHPLLAELLISINRVTRDDFENRSKLIDWIVRINKLSIGDTLTETQIRELLFDLELAYKSFYALLD
99	2O0Q	TLIYKILSRAEWDAAKAQGRFEGSAVDLADGFIHLSAGEQAQETAAKWFRGQANLVLLAVEAEPLGEDLKWEASRGGARFPHLYRPLLVSEVTREADLDLDADGVPQLGDHLAL
100	2O71	HILNSSPSDRQINQLAQRLGPEWEPMVLSLGLSQTDIYRCKANHPHNVQSQVVEAFIRWRQRFGKQATFQSLHNGLRAVEVDPSLLLHMLE

101	2OCH	KETGYYDVLGVKPDASDNELKKAYRKMALKFHPDKNPDGAEQFKQISQAYEVLSDEKKRQIYDQGG
102	2OUM	ALLEKVDPNKIYTIDEAAHLVKELATAKFDETVEVHAKLGIDPRRSDQNVRGTVSLPHGGRIEFRNDKTGAIHAPVGKASFPPEKLADNIRAFIRALEAHKPEGAKGTFLRSVYVTTTMGPSVRINPHS
103	2P5K	NKGQRHIKIREIITSNEIETQDELVDMLKQDGYKVTQATVSRDIKELHLVKVPTNNGSYKYSL
104	2PND	GHPTLKTPESVTGTWKGDVKIQCIYDPLRGYRQVLVKWLVRHGSDSVTIFLRDSTGDHIQQAKYRGRLKVSHKVPGDVSLQINTLQMDDRNHYTCEVTWQTPDGNQVIRDKIIELRVRK
105	2PST	DDIQFQVVVNHEEQYSIWPEYKEIPQGWRAAGKSGLKKDCLAYIEEVWTDMRPLSLRQHMD
106	2QVO	RIKLLFKEKALEILMTIYYESLGGNDVYIQYIASKVNSPHSYVWLIIKKFEEAKMVECELEGRTKIIRLTDKGQKIAQQIKSIIDIM
107	2QVT	GYTRFYRSPTASVTLSGLVNVKWDNEQMTMPLFKWIGGEQAEELHFCVHIAHSIGPKLNLARTLGTVNSNMDQHWAQAHRHSGATRRTIQDFHLFANDIPNFPDYIKIKLVPK
108	2RH3	IQVFLSARPPAPEVSKIYDNLILQYSPSKSLQMILRRALGDFENMLADGSFRAAPKSYPIPHTAFEKSIIVQTSRMFPVSLIEAARNHFDPLGLETARAFGHKLATAALACFFAREKATNS
109	2V75	YTENLKVIVAEKLAGIPNFNEDIKYVAEYIVLLIVNGGTVESVVDELASLFDSVSRDTLANVVQTAFFALEALQQGESAENIVSKIRMM
110	2V9V	GSPEKILAQIIQEHREGLDWQEAATRASLSLEETRKLLQSMAAAGQVTLLRVENDLYAISTERYQAWWQAVTRALEEFHSRYPLRPGLAREELRSRYFSRLPARVYQALLEEWSREGRLQLAANTVALAGFTPSF
111	2VC8	TDWLGSIVSINCGDSLGVYQGRVSAVDQVSQTISLTRPFHNGVKCLVPEVTFRAGDITELKILEIPGPGDNQ
112	2VO8	TTVGNSTIKVNDEVQVGSAFEAILGIEGLNGDTEVYSAEYLFEYNAEAFILNEITSFNDSLFVKSKEVEPGKVRILVASLGNEIEKDSDLVKVNLTPKISSELEVLGLTTALVGAGDGNTHDLELSSKEVKINEE
113	2W0G	HKTFVEKYEKQIKHFGMLRRWDDSQKYLSDNVHLVCEETANYLVIWCIDLEVEEKCALMEQVAHQTIVMQFILELAKSLKVDPRACFRQFFTKIKTADRQYMEGFNDELEAFKERVRGRAKLRIEKAMK
114	2W0I	MPLQPEAQRALQQLKQKMVNYIQMKLDLERETIELVHTEPTDVAQLPSRVPRDAARYHFFLYKHTHEGDPLESVVFIYSMPGYKCSIKERMLYSSCKSRLLDSVEQDFHLEIAKKIEIGDGAELTAEFLDDEVH
115	2Y6X	PTGLTGNFREDTLALISSLREAIALPENDPNKKAAQAEARKKLNDFFALYRRDDSLRSLSSFMTMQTALNSLAGHYSSYPNRPLPEKLKARLEQEFKQVELALDREAKS
116	2Y9F	MTDIYIPPEGLYFRLLGFASRQVIFARNSPSPDVGLSPVNDQATDQYFSLIYGTGEHAGLYAIKSKATGKVLFSRRPAEPYVGQIDGDGRYPDNWFKIEPGKTYLSKYFRLVQPSTGTALVSRTHLQPYFWNHPQTEVFDDQYFTFLFE
117	2YXY	EQKRYSEMTKEELQQEIAMLTEKARKAEQMGMVNEYAVYERKIAMAKAYMLNPADFHPGEIYEIEGAPGEYFKVRYLKGVFAWGWRLKGNGEEEALPISLLRKP
118	2ZQE	VKEVDLRGLTVAEALLEVDQALEEARALGLSTLRLLHGKGTGALRQAIREALRRDKRVESFADAPPGEGGHGVTVVALRP
119	2ZRR	GDDRRKKAEVIITELLDDLEIDLGNESLRKVLGSYLKKLKNEGTSVPLVLSRMNIEISNAIKKDGVSLNENQSKKLKELMSIS
120	3A4C	RCPEQELRLQRLERLPELARVLRNVFVSERKPALTMEVVCARMVDSCQTALSPGEMEKHLVLLAELLPDWLSLHRIRTDTYVKLDKAVDLAGLTARLAHHVHAEGL
121	3ADG	SHVFKSRLQEYAQKYKLPTPVYEIVKEGPSHKSLFQSTVILDGVRYNSLPGFFNRKAAEQSAAEVALRELAK
122	3AG7	GPLGSEEIKNIDAKIRKWSSGKSGNIRSLLSTLQYILWSGSGWKPVPLMDMIEGNAVRKSYQRALLILHPDKLQQKGASANQKYMAEKVFELLQEAWDHFNTLGP
123	3CE7	VVDTDINAVTNYIVGMCQKFLQKGEKVTPSSKLEELRTREDRLWDCLDTVEFVLDVEEIFDVTVPDEVADNFQTLQEIADFVVSERAKAG
124	3DJN	DMLPEIAAAVGFLSSLLRTRGCVSEQRLKVFSRALQDALTDHYKHHWFPEKPSKGSGYRCIRINHKMDPIISKVASQIGLSQPQLHRLLPSELTLWVDPYEVSYRIGEDGSICVLYEE
125	3DKM	GRENLYFQGLKYMVPGARVTRGLDWKWRDQDGSPQGEGTVTGELHNGWIDVTWDAGGSNSYRMGAEGKFDLKLAPGYDP
126	3E8V	AKGSVLVTDAEGQPVADATVEFKVYNYAEFYTVATKHTDRSGHASLTAGKGDMLVWASKDGRFGYSKLSFGKDNELKITLDK
127	3EGN	ECISRRELEKGRISREEMETLSVFRSYEPGEPNCRIYVKNLAKHVQEKDLKYIFGRYVDFSSETQRIMFDIRLMKEGRMKGQAFIGLPNEKAAAKALKEANGYVLFGKPMVVQFARSARP
128	3FBL	YHKLRLAIKEICKTDGIPNIKWGMYIAFGEKLLKSYLKMKAGSASSDMIAEYINNAISAFSSRTGISQETAQKIADFITSNY
129	3FDR	LQLDKLVNEMTQHYENSVPEDLTVHVGDIVAAPLPTNGSWYRARVLGTLENGNLDLYFVDFGDNGDCPLKDLRALRSDFLSLPFQAIEC
130	3FT9	VQVTFTVQKGSDPKKLVLDIKYTRPGDSLAEVELRQHGSEEWEPLTKKGNVWEVKSSKPLVGPFNFRFMSKGGMRNVFDEVIPTAFSIGKTYKP
131	3GK5	YRSINAADLYENIKAYTVLDVREPFELIFGSIANSINIPISELREKWKILERDKKYAVICAHGNRSAAAVEFLSQLGLNIVDVEGGIQSWIEEGYPVVLE
132	3HJ7	KGYWFELPVPALLPLPNGYAIISEFGEHYPRKQAGNDWFVVDPASVSLPLRVRTRRRGDRMVLKGTGGTKKLKEIFIEAKIPRMERDRWPIVEDADGRILWVPGLKKSAFEAQNRGQARYILLQYQAMNS
133	3HNX	HMSYADSSRNAVLTNGGRTLRAECRNADGNWVTSELDLDTIIGNNDGHFQWGGQNFTETAEDIRFHPKEGAAEQPILRARLRDCNGEFHDRDVNLNRIQNVNGRLVFQ
134	3I8Z	FAVESIEKKRIRKGRVEYLVKWRGWSPKYNTWEPEENILDPRLLIAFQNR
135	3ID1	MVRPVVGEIAANSIAAEAQIAPGTELKAVDGIETPDWDAVRLQLVDKIGDESTTITVAPFGSDQRRDVKLDLRHWAFEPDKEDPVSSLGIRPRG

136	3ID4	MIEPVLENVQPNSAASKAGLQAGDRIVKVDGQPLTQWVTFVMLVRDNPGKSLALEIERQGSPLSLTLIPESKPGNGKAIGFVGIEPKGKASPV
137	3IDW	KYDWFEFFLNCGVDVSNCQRYTINFDREQLTEDMMPDINNSMLRTLGLREGDIVRVMKHLDKKFGR
138	3IHO	KWTPPRSPFNLVQETLFHDPWKLLIATIFLNRTSGKMAIPVLWKFLEKYPSAEVARTADWRDVSELLKPLGLYDLRAKTIVKFSDEYLTKQWKYPIELHGIGKYGNDSYRIFCVNEWKQVHPEDHKLNKYHDWLWENH
139	3IU5	SMTVDPIAVCHELYNTIRDYKDEQGRLLCELFIRAPKRRNQPDYYEVVSQPIDLMKIQQKLKMEEYDDVNLLTADFQLLFNNAKSYYKPDSPEYKAACKLWDLYLRTRNEFVQK
140	3JU0	SLTDSKVKNAKSLEKEYKLTDGFGMHLLVHPNGSKYWRLSYRFEKKQRLLALGVYPAVSLADARQRRDEAKKLLAAGIDPSAKKQADNKTIQEKR
141	3JVE	APKPLHKVVVCVSKKLSKKQSELNGIAASLGADYRWSFDETVTHFIYQGRPNDTNREYKSVKERGVHIVSEHWLLDCAQECKHLPESLYPHTYN
142	3K3V	ESSWRYIDTQGQIHGPFTTQMMSQWYIGGYFASTLQISRLGSTPETLGINDIFITLGELMTKLEKYDTDPFTTFDKLHV
143	3KT9	MMRVCWLVRQDSRHQRIRLPHLEAVVIGRGPETKITDKKCSRQQVQLKAECNKGYVKVKQVGVNPTSIDSVVIGKDQEVKLQPGQVLHMVNELYPYIVEFEE
144	3L1X	SDAPDEFRDPLMDTLMTDPVRLPSGTIMDRSIILRHLLNSPTDPFNRQTLTESMLEPVPELKEQIQAWMREKQNS
145	3LF9	MINEIKKDAQERMDKSVEALKNNLSKVRTGGGGTEERRKDLVKIVRGEAEGGRVAVRNIARDAANDLAALGKDKEVNWFDISQALWEIQKLTDVAVKKIDEVLAAKEKELMEVLEHHHHH
146	3LLB	SYIKPLPSGDFIVKALTPVDAFNDFFGSEFSDEEFDTVGGLVMSAFGHLPKRNEVVELGEFRFRVLNADSRRVHLLRLSPL
147	3LMO	TSTFDRVATIIAETCDIPRETITPESHAIDDLGIDSLDFLDIAFAIDKAFGIKLPLEKWTQEVNDGKATTEQYFVLKNLAARIDELVAAKGALE
148	3MX7	MTDLVAVWDVALSDGVHKIEFEHGTTSGKRVVYVDGKEEIRKEWMFKLVGKETFYVGAAKTKATINIDAISGFAYEYTLEINGKSLKKYM
149	3NDQ	RAPSTSDSVRLKCREMLAAALRTGDDYIAIGADEEELGSQIEEAIYQEIRNTDMKYKNRVRSRISNLKDAKNPNLRKNVLCGNIPPDLFARMTAEEM
150	3NE3	GIVGVGIDLVSIPDFAEQVDQPGTVFAETFTPGERRDASDKSSSAARHLAARWAAKEAVIKAWSGSRFAQRPVLPEDIHRDIEVVTDMWGRPRVRLTGAIAEYLADVTIHVSLTHEGDTAAAVAILEAP
151	3NPH	ELRSRSTEEEVDAVILAVYRQVLGNDHLMSQERLTSAESLLRGREISVRDFVRAVALSEVYRQKFFHSNPQNRFIELNYKHLLGRAPYDQSEIAFHTDLYHQGGYEAEINSYIDSVEYTENFGDWVVPYFR
152	3O48	DFWPTLKDAYEPLYPQQLEILRQQVVSEGGPTATIQSRFNYAWGLIKSTDVNDERLGVKILTDIYKEAESRRRECLYYLTIGCYKLGEYSMAKRYVDTLFEHERNNKQVGALKSMVEDKIQKEENLYFQ
153	3O5E	SPTATVAEQGEDITSKKDRGVLKIVKRVGNGEETPMIGDKVYVHYKGKLSNGKKFDSSHDRNEPFVFSLGKGQVIKAWDIGVATMKKGEICHLLCKPEYAYGSAGSLPKIPSNATLFFEIELLDFKGE
154	3OBS	SAVSESQLKKMVSKYKYRDLTVRETVNVITLYKDLKPVLDSYGTGSRELMNLTGTIPVPYRGNTYNIPICLWLLDTYPYNPPICFVKPTSSMTIKTGKHVDANGKIYLPYLHEWKHPQSDLLGLIQVMIVVFGDEPPVFS
155	3ONH	KVCRGVIKLSSDCLNKMKLSDFVVLIREKYSYPQDISLLDASNQRLLFDYDFEDLNDRTLSEINLGNGSIILFSDEEGDTMIRKAIELFLDVDDELPCNTCSLPDVEVPLIKA
156	3ONJ	SLLISYESDFKTTLEQAKASLAEAPSQPLSQRNTTLKHVEQQQDELFDLLDQMDVEVNNSIGDASERATYKAKLREWKKTIQSDIKRPLQSLVDSGD
157	3PDG	MVTIDSPVAGERFEAGKDINISATVKSKTPVSKVEFYNGDTLISSDTTAPYTAKITGAAVGAYNLKAVAVLSDGRRIESPVTPVLVKVIVL
158	3QD7	DTLQLDNFLTTGFLDIIPLSQPLEFRREGLQHGVLDKLRSGKYPQQASLNLLRQPVEECRKMVFSFIQQALADGLRNVLIIHGKGRDDKSHANIVRSYVARWLTEFDDVQAYCTALPHHGGSGACYVALRK
159	3RJ2	IVANCEFVNATGKKTTILVNENWAKYCWIWTYKFPEKYTLLRYSVDGEMFMRHRVTFFNATGRYITHTHLNHGLEDVLEGSLAVPKDAAYARIHAAINVSLTNPGDVHMHYDETEGEQIRSYDAAEFARTLAA
160	3RJP	MYRDLVLNPQNRSVNRGDDEISLTKREYDLLNILMTNMNRVMTREELLSNVWKYDEAVETNVVDVYIRYLRGKIDIPGKESYIQTVRGMGYVIREK
161	3T46	HQALVDQLHELIANTDLNKLSYLNLDAFQKRDILAAHYIAKSAIRTKNLDQMTKAKQRLESIYNSISNPLHSQNN
162	3V4Q	LAAKEAKLRDLEDSLARERDTSWRLLAEKEREMAEMRARMQQQLDEYQELLDIKLALDMEIHAYRKLLEGEEER
163	3ZHI	KTDTSNRLKQIMAERNLKQVDILNLSIPFQKKFGIKLSKSTLSAYVASVQSPDQNRIYLLAKTLGVSEAWLMG
164	3ZZP	QGYTTWYQVEMPEDRVNDLARELRIRDNVRRVMVVASTTPGRYEVNIVLNPNLDQSQLQNEKEIIQRALENYGA
165	4EIH	SLEKHSWYHGPVSRSAAEYLLSSLINGSFLVRESESSPGQLSISLRYEGRVYHYRINTTADGKVYVTAESRFSTLAELVHHHSTVADGLVTTLHYPAPKCN
166	4F25	SGNIFIKNLDKSIDNKALYDTFSAFGNILSCKVVCDENGSKGYGFVHFETQEAAERAIEKMNGMLLNDRKVFVGRFKSRKE
167	4FI5	SMTMEELQREINAHEGQLVIARQKVRDAEKQYEKDPDELNKRTLTDREGVAVSIQAKIDELKRQLADRIAT
168	4G3O	RGSENLYFQGQLNAMAHQIQEMFPQVPYHLVLQDLQLTRSVEITTDNILEGRI
169	4GA2	RRSKADVERYIASVQGSTPSPRQKSIKGFYFAKLYYEAKEYDLAKKYICTYINVQERDPKAHRFLGLLYELEENTDKAVECYRRSVELNPTQKDLVLKIAELLCKNDVTDGRAKYWVERAAKLFPGSPAVYKLKEQLLDCEGED
170	4GCO	YINPELAQEEKNKGNEYFKKGDYPTAMRHYNEAVKRDPENAILYSNRAACLTKLMEFQRALDDCDTCIRLDSKFIKGYIRKAACLVAMREWSKAQRAYEDALQVDPSNEEAREGVRNCLR

171	4GEI	KIKSFEVVFNDPEKVYGSGERVAGRVIVEVSEVTRVKAVRILASGVAKVLWMQGSQQCAQTSEYLRYEDTLLLEDQPTGENEMVIMRPGNKYEYKFGFELPQGPLGTSFKGKYGSVDYWVKAFLDRPSQPTQETKKNFEVVDLV
172	4GMQ	NAVKKNKRVLRGSVKEANYFVEGEASAATIDAVLNDVDLVITKIDADEIAALAGKLNGLTVADEIKNVWKEEVSRLVGAGKLKEGDIKALVA
173	4HQA	SRKFIIANARVENCAVIYCNDGFCELCGYSRAEVMQRPCTCDFLHGPRTQRRAAAQIAQALLGAEERKVEIAFYRKDGSCFLCLVDVVPVKNEDGAVIMFILNFEVVMEK
174	4HTJ	ARGYIVTDRDPLRPEEGRRLVEDVARLLQVPSSAFADVEVLGPAVTFKVSANVQNVTTEDVEKATVDNKDKLEETSGLKILQTGVGSKS
175	4IGV	DLSGKMVKQVEILSDGIVFYEIFRYRLYLISEMSPVNIQGVDLLEGNWGTVGSVIFFKYTIDGKEKTAKDIVEAIDEETKSVTFKIVEGDLMELYKTFIIIVQVDTKGEHNSVTWTFHYEKLKEDVEEPNTLMNFCIEITKDIETYHLK
176	4IPC	GSHMVGQLSRGAIAAIMQKGDTNIKPILQVINIRPITTGNSPPRYRLLMSDGLNTLSSFMLATQLNPLVEEEQLSSNCVCQIHRFIVNTLKDGRRVVILMELEVLKSAEAVGVKIGNPVPYNE
177	4J5Q	EDPEGSRITYVKGDLFACPKTDSLAHCISEDCRMGAGIAVLFKKKFGGVQELLNQQKKSGEVAVLKRDGRYIYYLITKKRASHKPTYENLQKSLEAMKSHCLKNGVTDLSMPRIGCGLDRLQWENVSAMIEEVFEATDIKITVYTL
178	4JOX	AREVHIDVNNKTGHTLQLEDKTKLDGGRWRTSPTNVANDQIKTFVAESNGFMTGTEGTIYYSINGEAEISLYFDNPFAGSNKYDGHSNKSQYEIITQGGSGNQSHVTYTIQTTSSRYG
179	4KMC	TNWSRRYKANLEKLASGDVNKVAEVVRDLWRRDQERGLSAGEKRMLAKARQILVGELALAESTDDAKAETILDEVLAAA
180	4LTT	MLKLNLKKSFQKDFDKLLLNGFDDSVLNEVILTLRKKEPLDPQFQDHALKGKWKPFRECHIKPDVLLVYLVKDDELILLRLGSHSELFLEG
181	4MNO	VIRVPLPEGNQLFGVVEQALGAGWMDVRCEDGKIRRCRIPGKLRRRVWIRVGDLVIVQPWPVQSDKRGDIVYRYTQTQVDWLLRKGKITQEFLTG
182	4MQ3	MGIWQMDCTHFDGKIILVGIHVESGYIWAQIISQETADCTVKAVLQLLSAHNVTELQTDNGPNFKNQKMEGVLNYMGVKHKFGIPGNPQSQALVENVNHTLKVWIQKFLPETTSLDNALSLAVHSLNKK
183	4N1L	SSFFPDFGLLLYLEELNKEELNTFKLFLKETMEPEHGLTPWNEVKKARREDLANLMKKYYPGEKAWSVSLKIFGKMNRKDLCERAKEEINWSAQL
184	4N6T	ENSLEIEELARFAVDEHNKKENALLEFVRVVKAKEQVVAGTMYYLTLEAKDGGKKKLYEAKVWVKPWENFKELQEFKPV
185	4NPN	HINLKVAGQDGSVVQFKIKRHTPLSKLMKAYCERQGLSMRQIRFRFDGQPINETDTPAQLEMEDEDTIDVF
186	4O7Q	AMESKYKEILLLTGLDNITDEELDRFKGFLSDEFNIATGKLHTANRIQVATLMIQNAGAVSAVMKTIRIFQKLNYMLLAKRLQEEKEKVDKQYK
187	4O9L	PHMTVAEDKTFQYIRQHHSNFSRIHVLRILPYLSCLTTSDQDRLRATYERWGNQDTLLELFTSLRRRNGWVHSLIGALRACELSGLADEVARIYHS
188	4OD6	RFDNELQLRGLSVEAAVEELRAAIAEARALKETPLRVVHGKGMGVLRRTLRDYLKTDKNVESFHDAEANQGGHGVTIVNVKR
189	4OIX	MLKRMYARVYGLVQGVGFRKFVQIHAIRLGIKGYAKNLPDGSVEVVAEGYEEALSKLLERIKQGPPAAEVEKVDYSFSEYKGEFEDFETY
190	4PGO	EILSKKPAGKVVVEEVVNIMGKDVIIGTVESGMIGVGFKVKGPSGIGGIVRIERNREKVEFAIAGDRIGISIEGKIGKVKKGDVLEIYQT
191	4Q2S	IAESLRRLKEYVKAGSVKECVAEWCNMPSVAGFDVLSEISYDRMLENCSNLLLLTFIYHISLLDSVDDDRLSKRMEYISRICLNIDVNDPKVETVVHPVLTLTREALLRQSEFFSPIFKRRLVVLLRALDGKISEI
192	4Q6L	YDWNGAMQPLVSKMLQADGVTAGSVLLVDSVNNRTNGSLNANEATETLRNALANNGKFTLVSVQQLSMAKQQLGLSPQDSLGTRSKAIGIARNVGAQYVLYSSASGNVNAPALQMQLMLVQTGEIIWSGKGAVQQ
193	4QB0	EHSFEEMYRHILRSQGPFDAVLYYHMMKDEPVVFSTSDGKEYTYPDSLEEEYPPWLTEKEAMNEENRFVTLDGQQFYWPVMNHKNKFMAILQHHQ
194	4QCJ	LPAGSALLVVKRGPNAGARFLLDQPTTTAGRHPESDIFLDDVTVSRRHAEFRINEGEFEVVDVGSLNGTYVNREPRNAQVMQTGDEIQIGKFRLVFLAGPA
195	4QUC	EYVVEKILGKRFVNGRPQVLVKWSGFPNENNTWEPLENVGNCMKLVSDFESEVFRL
196	4RWU	MVKETKLYDLLGVSPSANEQELKKGYRKAALKYHPDKPTGDTEKFKEISEAFEILNDPQKREIYDQYGLEAARSGGPSFGP
197	4W8G	TYDVNICYAPEDREWVINTLVFKLERAGIKTFVNIRDDTPGNFFAENIMDAIENSNRTIVVMSPDFFKNNICDKTLQIGLSHQIIPILYRPCEVPYFLNHMTYLDWCDKDVRPVFWRNLFRDIRN
198	4YKD	ASATELLQDYMLTLRTKLSSQEIQQFAALLHEYRNGASIHEFCINLRQLYGDSRKFLLLGLRPFIPEKDSQHFENFLETIGVKD
199	4ZC3	LSPKQERFIEEYFINDMNATKAAIAAGYSKNSASAIGAENLQKPAIRARIDARLKEI
200	5CVZ	AAATSLVYDTCYVTLTERATTSFQRQSFPTLKGMGDRAFQVVAFTIQGVSAAPLMYNARLYNPGDTDSVHATGVQLMGTVPRTVRLTPRVGQNNWFFGNTEEAETILAIDGLVSTKGANAPSNTVIVTGCFRLAPSELQSS
201	5CW9	MEMDIRFRGDDPEAYYKALREMIRQARKFAGTVTVTLIIRFRGDDLEALEKALKEMIRQARKFAGTVTYTLDGNDLEIRITGVPPQVILELVKEAIRLAKEFNITVTVELVIRITGVPEQVRKELAKEAERLAKEFNITVTYTIRL
202	5DBL	HMIAPGHRDEFDPKLPTGEKEEVPGKPGIKNPETGDVVRPPVDSVTKYGPVKGDSIVEKEEIPFEKERKFNPDLAPGTEKVTREGQKGEKTITTPTLKNPLTGEIISKGESKEEITKDPINELTEWGPET
203	5DMA	GWKVGDRANHRKWGIGTVVSVRGGGDDQELDIAFPSPIGIKRLLAKFAPIEKV
204	“5E10”	GVQVPDVRGQSSADAIATLQNRGFKIRTLQKPDSTIPPDHVIGTDPAANTSVSAGDEITVNVSTGPEQREIPDVSTLTYAEAVKKLTAAGFGRFKQANSPSTPELVGKVIGTNPPANQTSAITNVVIIIVGSG
205	5E1Y	SMEILQVALHKRDSGEQLGIKLVRRTDEPGVFILDLLEGGLAAQDGRLSSNDRVLAINGHDLKYGTPELAAQIIQASGERVNLTIARPGKPEIEL

206	5EEP	GHPFGTTVTAETLRNTFAPLTQWEDKYRQLIMLGKQLPALPDELKAQAKEIAGCENRVWLGYTVAENGKMHFFGDSEGRIVRGLLAVLLTAVEGKTAAELQAQSPLALFDELGLRAQLSASRSQGLNALSEAIIAAAKQV
207	5EFX	GARLQEIYNRMDPRAQTPVPGKGPFGREELLRRKLIHDGCLLWKTATGRFKDVLVLLMTDVLVFLQEKDQKYIFPTLDKPSVVSLQNLIVRDIANQEKGMFLISAAPPEMYEVHTASRDDRSTWIRVIQQSVRTCPSREDFPLI
208	5EJO	AMITDPMDLLRLFDGVQDSTFSLGTVTEIAQKNLPQYNKQTIKNTIKEYAIRSSGKGDLPRKWVIKDAQNWENLRANAN
209	5EQZ	HHHYVEEKKEIDSLMEDVLALVNDSSGGKFKDYKDKINELKENLKDIGNAELKEKLLNLQNSFQDKLAAKLAALKAAKNTIENITDKDQDISKRKIWSEAKLVGVTVPLLGSNTSGNGDKMSKNAVEQIDKVIKFLEE
210	5EWR	DPRAYPFAPADLVVEILDLVQQASHYKQIKKGLNEVLKSMNRGLAEFVVLAADTQPLEILLSAPLVAEDKAVPYVFVPSKAALGRACGVSRPVIACAVLRADMSQLRNQITALRTKIEQLLL
211	5FD9	SPTKAIVRLREHINLLSKKQSHLRTQITNQENEARIFLTKGNKVMAKNALKKKKTIEQLLSKVEGTMESMEQQLFSIESANLNLETMRAMQEGAKAMKTIHSGLDIDKVDETMDEIREQVELGDE
212	5FU5	GPVAGNYVHDFTANGTSSSFYTIAGNLSTSKGTATYNGKTLTQCLKMETATSISFTAPSAGKLTLVFAEAAATAKVDGNKVTASNGIITVDLAQGAHTITKADACNLFYMEYAALEH
213	5GUA	MENVVSAPMPGKVLRVLVRVGDRVRVGQGLLVLEAMKMENEIPSPRDGVVKRILVKEGEYVDTGQPLIELG
214	5H1D	PNVVVTRLTLVCESAPGPITMDLTGDLEALKKETIVLKEGSEYRVKIHFKVNRDIVSGLKYVQHTYRTGVKVDKATFMVGSYGPRPEEYEFLTPVEEAPKGMLARGTYHNKSFFTDDDKQDHLSWEWNLSIKKEW
215	5HBQ	TTISPHDAQELIARGAKLIDIRDADEYLREHIPEADLAPLSVLEQSGLPAKLRHEQIIFHDQAGKRTSNNADKLAAIAAPAEIFLLEDGIDGWKKAGLPVAVN
216	5HDW	SEFVATTGDITVSVSTSFLPELSSVHPPHYFFTYRIRIEMSKDALPEKACQLDSRYWRITNAKGDVEEVQGPGVVGEFPIISPGRVYEYTSCTTFSTTSGYMEGYYTFHFLYFKDKIFNVAIPRFHMACPT
217	5HFD	GSPEFLGEEDIPREPRRIVIHRGSTGLGFNIVGTEDGEGIFISFILAGGPADLSGELRKGDQILSVNGVDLRNASAEQAAIALKNAGQTVTIIAQYKPEEYSRFEANSRVDSSGRIVTD
218	5HU4	DAVVGSIAVPSVDVNLLVFKGTNTANLLAGATTMRSDQVMGKGNYPLAGHHMRDESMLFGPIMKVKKGDKIYLTDLENLYEYTVTETKTIDETEVSVIDNTKDARITLITCDKPTETTKRFVAVGELEKTEKLTKELENKYFPSK
219	5I41	SHMNTNMVASELGVSAKTVQRWVKQLNLPAERNELGHYSFTAEDVKVLKSVKKQISEGTAIQDIHLP
220	5IJM	SHMSAADFEAAVAYVRSLPKEGPVQLDNAAKLQFYSLYKQATEGDVTGSQPWAVQVEARAKWDAWNSCKGMKSEDAKAAYVRRLLTLLRSQGIQWKPG
221	5IWH	GIKSFKHKGLKLLFEKGVTSGVPAQDVDRINDRLQAIDTATEIGELNRQIYKLHPLKGDREGYWSITVRANWRITFQFINGDAYILNYEDYKLGPEQ
222	5JBM	QKAMITDPMDLLRLFDGVQDSTFSLGTVTEIAQKNLPQYNKQTIKNTIKEYAIRSSGKGDLPRKWVIKDAQNWENLRANAN
223	5JRT	VDFSITQFVRNLGLEHLMDIFEREQITLRVLVEMGHKELKEIGINAYGHREKLIKGVERLISGQ
224	5KIU	HHQKLSARLRALRQRQLDRAAAAVEPDVVVKRQEALAAARLKMQEELNAQVEKHKEKLKQLEEEKRRQKIEMWDSM
225	5KUX	GIVNVPNCNTTKYQQLARTAVAIYNYHEQAHLTFVENLNCKEQLGEGDYYYITLAATDDAGKKAIYEAKIGVVESAGWTGVEEFKLVGSLEH
226	5L33	HMPEEEKAARLFIEALEKGDPELMRKVISPDTRMEDNGREFTGDEVVEYVKEIQKRGEQWHLRRYTKEGNSWRFEVQVDNNGQTEQWEVQIEVRNGRIKRVTITHV
227	5ML3	SAKDERAREILRGFKLNWMNLRDAETGKILWQGTEDLSVPGVEHEARVPKKILKCKAVSRELNFSSTEQMEKFRLEQKVYFKGQCLEEWFFEFGFVIPNSTNTWQSLIEAAPESQMMPASVLTGNVIIETKFFDDDLLVSTSRVRLFYV
228	5MR1	ADCQGWLYKKKEKGSFLSNKWKKFWVILKGSSLYWYSNQMAEKADGFVNLPDFTVERASECKKKHAFKISHPQIKTFYFAAENVQEMNVWLNKLGSAVIHQ
229	5NOD	VTSVAMPSYIGSSLEFTKNNLIQIVGIKEANIEVVEVTTAPAGSVEGMVVEQSPRAGEKVDLNKTRVKISIYKPK
230	5OHQ	GPWVTTDIQVKVRDTYLDTQVVGQTGVIRSVTGGMCSVYLKDSEKVVSISSEHLEPITPTKNNKVKVILGEDREATGVLLSIDGEDGIVRMDLDEQLKILNLRFLGKLLE
231	5TPJ	MSREEIRKVVEEMVRKLKQGSPEDISKYLSPDVRLEVGNYTFEGSEQVTKFWRMLTKFVDRVEVRKVQVDGNHVRVEVEVEWNGKKWTFEVEVEVRNGKIKRIRLQVDPEFKKVVQNIWNLL
232	5UFT	SMIDDIYNKRILEFAGNMERIGQLAEPDAVATVHSKLCGSTVTVYLKMRDGVVTDFAHEVKACALGQASSSVMARNVIGATADELRAARDAMYRMLKENGPAPEGRFADMKYFEPVRDYKARHASTLLTFDAVADCIRQIEEKA
233	5UMV	SSKIFKNCVIYINGYTKPGRLQLHEMIVLHGGKFLHYLSSKKTVTHIVASNLPLKKRIEFANYKVVSPDWIVDSVKEARLLPWQNYSLTS
234	5WD9	APVTELTRLKEYMEDQIAKAKESSSLTAQLKFLENAHTEHFVKMGSLTTIYKGGSEVVDRLKIEIRSLYEEMLELKDKCRDQIQQY
235	5WF2	AMTLSDCQQELSLVQTVTRGSRAFLSREEAQHFVKECGLLNCEAVLELLICHLGGTSECVQMRALGAVASLGCTDLLPQEHILLLTRPRLQELSAGSPGPVTNKATKILRHFEASC
236	5XUO	AGFGAQHRKMGKSLYLRNEVFAAWIEKVDALVQDELGYSVLELILDDAQDYGIETTQVTIFAIQIALGELLRHHGAKPAAVIGQSLGEAASAYFAGGLSLRDATRAICSRS
237	5Y3C	PAAASTKVLYYTDRSLTPFLVNIPKRLGDVTLQDFKAAVDRHGSFRYHFKSLDPEFGTVKEEVFQDDAVIPGWEGKIVAWVEE
238	5Y4U	ARLTKLVNAAPVMLFMKGSPSEPKCGFSRQLVGILREHQVRFGFFDILRDESVRQNLKKFSEWPTFPQLYINGEFQGGLDIIKESLEEDPDFLQHALQS
239	5Y6H	YHEQFLKQNPLAVLGVLRDLHKAAIPLRLSWNGGQLISKLLAITPDKLVLDFGSQAEDNIAVLKAQHITITAETQGAKVEFTVEQLQQSEYLQLPAFITVPPPTLWFVLE
240	5YDE	MADVLSVLRQYNIQKKEIVVKGDEVIFGEFSWPKNVKTNYVVWGTGKEGQPREYYTLDSILFLLNNVHLSHPVYVRRAATENIPVVRRPDRKDLLGYLNGEASTSASIDRS

241	5YDN	MNYATVNDLCARYTRTRLDILTRPKTADGQPDDAVAEQALADASAFIDGYLAARFVLPLTVVPSLLKRQCCVVAWFYLNESQPTEQITATYRDTVRWLEQVRDGKTDPG
242	5YRY	PEFMENLVTLSRQGIENLMKLENRKEPWIVVLYAPWCPFCQAMEASYDELADKLAGSGIKVAKFRADGDQKEFAKQELQLGSFPTILVFPKNSSRPIKYPSEKRDVESLTSFLLEH
243	5YWJ	LLGFYKQYKALSEYIDKKYKLSLNDLAVLDLTMKHCKDEKVLMQSFLKTAMDELDLSRTKLLVSIRRLIEKERLSKVRSSKDERKIYIYLNNDDISKFNALFEDVEQFLN
244	5Z59	WPSAKFIDGRVAFSRMPAERELDEVARDFDAVVVLVEDYELPYSLDEWEKRGVEVLHGPIPDFTAPSVEQLLEILRWIEERVREGKKVLIHCMGGLGRSGTVGVAWLMYSRGLSLREALMEVRRKRPGAVETQEQMEVLKELEERI
245	5ZT3	YPYPVDEIIGGDSVQSIQRRLLGTNWNPSAHDMQMSRIQAEDLFELKVEIIRKMAGLHPSGDWMGWGARALDNPRTATGEEDLARLHQMLDDLQSRNEQSATFWRLVERVRLRA
246	6AR0	PSALAIFTCRPNSHPFQERHVYLDEPIKIGRSVARCRPAQNNATFDCKVLSRNHALVWFDHKTGKFYLQDTKSSNGTFINSQRLSRGSEESPPCEILSGDIIQFGVDVTENTRKVTHGCIVSTIKLFLPDGMEARLRS
247	6B26	NSYVALYKFLPQENNDLALQPGDRIMLVDDSNEDWWKGKIGDRVGFFPANFVQRVRPGENVWRCCQPFSGNKEQGYMSLKENQICVGVGRSKDADGFIRVSSGKKRGLVPVDALTEI
248	6B8C	VNGAAIVAEAYKYIGTPYVWGGKDPSGFDCSGFTRYVYLQVTGRDIGGWTVPQESAGTKISVSQAKAGDLLFWGSPGGTYHVAIALGGGQYIHAPQPGESVKVGSVQWFAPDFAVSM
249	6BL5	RVPRLHAYGVFALPFPMDPDVEWGNWFAGPHPKAFLVSVHPSGPKAGHVYPTDLSDPDSVANVIGMVLDGHDYEADHNVTVTLRAAVPIEYVQQGIEAPPLQPDPAVLNAAPQLKLKVIKGHYFFDYTR
250	6D1R	KAYRIKKNADFQRIYKKGHSVANRQFVVYTCNNKEIDHFRLGISVSKKLGNAVLRNKIKRAIRENFKVHKSHILAKDIIVIARQPAKDMTTLQIQNSLEHVLKIAKVFN
251	6DGA	MKDDLDNNLLYRYCGATSPFWRLPLDSNALQLAASEEAVTSHVVPLTPEQAAQIRTMSVITSSVTLSLSLFGELVPVHLVGRKVSRKEWAGTASA
252	6E4N	GSHMRVQVSGMSDETTWHTLKDHLRQAGEVTFCKVFSGGRAVVEFVTPEDAARAITELQASELEGATLFLR
253	6ELM	SMAEDTGVRVELAEEDHGRKSTIALRLWVEDPKKLKGKPKDNGAIEFTFDLEKETPDEVAQEMIESGFFHESDVKIVAKSIRDRVALIQWRRERIWPA
254	6EW6	DSQIQFTRHASDVLLNLNRLRSRDILTDVVIVVSREQFRAHKTVLMACSGLFYSIFTDQLKRNLSVINLDPEINPEGFNILLDFMYTSRLNLREGNIMAVMATAMYLQMEHVVDTARKFIKAS
255	6EXX	KISTLFLENLSAVCNKEFLKYLCHQENIRPFKIQIDGYDENSSTYSGFIKFRNFEDATRIFNFLNNRLVGGSIVTTSWE
256	6F53	LPTAQEVQGLMARYIELVDVGDIEAIVQMYADDATVEDPFGQPPIHGREQIAAFYRQGLGGGKVRACLTGPVRASHNGCGAMPFRIEMVWNGQPCAVDAISVMRFDEHGRIQTMQAYWSEVNLSV
257	6FC5	GPMDRYQRKIGCFIQIPNLGRGQLKYVGPVDTKAGMFAGVDLLANIGKNDGSFMGKKYFQTEYPQSGLFIQLQKVASLIEKASI
258	6GPM	GQVIVVRKDSDFRPKTYEDLVGKTVAVQIGTTGDIEVSKYDGIKVVRFDKFTDAFLELKRGRADAVVLDSATARAFVAKNPDLVISSGVLSS
259	6IPY	TGVTLFVALYDYEAITEDDLSFHKGEKFQILNSSEGDWWEARSLTTGETGYIPSNYVAPVDSILEHH

Disprot dataset

**#**	**Disprot II**	**Sequence**
1	DP00070	SKTKEGVVHGVATVAEKTKEQVTNVGGAVVTGVTAVAQKTVEGAGSIAAAT
2	DP00284	TNVLNQGGEVGYDSSSNVIQDSNLDTAINPDDRPLSEIPTDDNPNMSMAEM
3	DP00284	YGVEVKTPARSLSAMAATVAKSSDPADPPIPNDSRILFKEPVSSYKGEYPY
4	DP00088	MFGKLSLDAVPFHEPIVMVTIAGIILGGLALVGLITYFGKWTYLWKEWLTS
5	DP00262	SDLACRLLGQSMDESGLPQLTSYDCEVNAPIQGSRNLLQGEELLRALDQVN
6	DP00974	MDSSAGPGNISDCSDPLAPASCSPAPGSWLNLSHVDGNQSDPCGPNRTGLG
7	DP00808	MEDINFASLAPRHGSRPFMGNWQDIGTSNMSGGAFSWGSLWSGIKNFGSTV
8	DP00488	MEAHNQFLKTFQKERHDMKEAEKDEILLMENSRRFVMFPIKYHEIWAAYKK
9	DP00226	EKPSYDTEADPSEGLMNVLKKIYEDGDDDMKRTINKAWVESREKQAREDTEF
10	DP00282	NIEGLLHKNNWNIEDNNIKNASLVQIDASNEQSSTSSSMIIDAQISNALNAQ
11	DP00623	SNHTFYPWMAIAGECPEDPTKSKIRSDLTQYGGISTDMGKRYSESLAGSLLP
12	DP00807	PPGATPAPPGAAPPPAAGGSPSPPADGGSPPPPADGGSPPVDGGSPPPPSTH
13	DP00875	KVGSLQYLALAALITPKKIKPPLPSVTKLTEDRWNKPQKTKGHRGSHTMNGH

14	DP00793	ASVPAGGAVAVSAAPGSAAPAAGSAPAAAEEKKDEKKEESEESDDDMGFGLFD
15	DP00298	SDVEENRTEAPEGTESEMETPSAINGNPSWHLADSPAVNGATGHSSSLDAREV
16	DP00624	GKQRMKEKEQENERKVAQLAEENERLKQEIERLTREVEATRRALIDRMVNLHQ
17	DP01080	CQSGSTCVKQNDWYSQCLPGSQVTTTSTTSTSSSSTTSRATSTTRTGGVTSIT
18	DP01048	NRESARRSRYRKAAHLKELEDQVEQLKAENSCLLRRLAALNQKYNEANVDNRV
19	DP00061	VPYNEGLGQPQVAPPAPAASPAASSRPQPQNGSSGMGSTVSKAYGASKTFGKA
20	DP00224	MHHQQRMAALGTDKELSDLLDFSAMFSPPVSSGKNGPTSLASGHFTGSNVEDR
21	DP00037	RARMESGQYRCLDWWFCWDTPASRDDVEEARRYLRRAAEKPAKLLYEEARTHLH
22	DP00558	MAATEGVGEAAQGGEPGQPAQPPPQPHPPPPQQQHKEEMAAEAGEAVASPMDDG
23	DP00835	MSQRIIQPSASDQQFPGKSDGYEYTVGPKQAITSEASTTYIPSRIYSESLLFKR
24	DP00028	MSGGSSCSQTPSRAIPATRRVVLGDGVQLPPGDYSTTPGGTLFSTTPGGTRIIYD
25	DP00148	MQRGNFRNQRKMVKCFNCGKEGHTARNCRAPRKKGCWKCGKEGHQMKDCTERQAN
26	DP00424	TYLGRSAEPVPLQLPPLERLTLDCNEDCGTSGTQGVGSPQILVESPTILESGAKE
27	DP00427	MSERFPNDVDPIETRDWLQAIESVIREEGVERAQYLIDQLLAEARKGGVNVAAGT
28	DP00926	GWQAFKNDATEIIPELGEYPEPPPELENNKTMNRAENGGRPPHHPFETKDVSEYS
29	DP00092	ITSFEEAKGLDRINERMPPRRDAMPSDANLNSINKALTSETNGTDSNGSNSSNIQ
30	DP00194	DEDTSIMNDEEASADNETVMSVIDGDKPDHDDDTHPDDEPPQPPRGGRPALRVVK
31	DP00506	KNRKAKAKPVTRGAGAGGRQRGQNKERPPPVPNPDYEPIRKGQRDLYSGLNQRRI
32	DP00600	QGTRAQKTRAEVTGSGKKPWRQKGTGRARSGSIKSPIWRSGGVTFAARPQDHSQKV
33	DP00748	IYARVIQKRVPNAYDKTALALEVGELVKVTKINVSGQWEGECNGKRGHFPFTHVRL
34	DP00048	GGKWSKSSVVGWPTVRERMRRAEPAADGVGAASRDLEKHGAITSSNTAATNAACAW
35	DP00224	MHHQQRMAALGTDKELSDLLDFSAMFSPPVSSGKNGPTSLASGHFTGSNVEDRSSS
36	DP00175	MPQLNGGGGDDLGANDELISFKDEGEQEEKSSENSSAERDLADVKSSLVNESETNQ
37	DP00769	EEERPRRQFNKGGRTGERDGRNKRGVGRGTRREGEEAAKEEGAAETAQGNQETPAQE
38	DP01015	ELKITETEPGVRGDTATGGTKRAKLETGAVVRVPLFLNEGEIIKVDTRRGEYVSRAK
39	DP00083	SDPAALKRARNTEAARRSRARKLQRMKQLEDKVEELLSKNYHLENEVARLKKLVGER
40	DP00027	VTLSDAQAKLMQPGVSDINMERVEALKTAIRNGELKMDTGKIADSLIREAQSYLQSK
41	DP00948	PTEPKKDKKKKTDEAQPLPQRQKKQPTVTLLPAADMDDFSRQLQNSMSGASADSTQA
42	DP00949	DKPTPEEHGPGIDIFTPGKPGEYDLEGGIWEDEDARNFYENLIDLKAFVPAILFKDN
43	DP00287	MPRRAENWDEAEVGAEEAGVEEYGPEEDGGEESGAEESGPEESGPEELGAEEEMEAG
44	DP01073	ASTASPAKAESSGVIDLFGDAFGSGASETQPAPQAVSSSSASADLLAGFGGSFMAPST
45	DP00803	MASLATLAAVQPTTLKGLAGSSIAGTKLHIKPARQSFKLNNVRSGAIVAKYGDKSVYF
46	DP00111	GKLGGQDSFESVESYDSCDRLTQSWSSQSSFNSLQRVPSYDSFDYEDYPAALPNHKPK
47	DP00750	AQSEQRDRFIPSYEETQRILKEQAEAEEAARNDPDLQAAQEENMRKIREMLGINRGGAR
48	DP00600	AQKTRAEVTGSGKKPWRQKGTGRARSGSIKSPIWRSGGVTFAARPQDHSQKVNKKMYRG

49	DP00348	PPRSISPSALQDLLRTLKSPSSPQQQQQVLNILKSNPQLMAAFIKQRTAKYVANQPGMQ
50	DP00249	MFDKNADGYIDLEELKIMLQATGETITEDDIEELMKDGDKNNDGRIDYDEFLEFMKGVE
51	DP00348	PPRSISPSALQDLLRTLKSPSSPQQQQQVLNILKSNPQLMAAFIKQRTAKYVANQPGMQP
52	DP01053	MPKSKELVSSSSSGSDSDSEVDKKLKRKKQVAPEKPVKKQKTGETSRALSSSKQSSSSRD
53	DP00975	YPERDSAKEGNQEQERALHVKVQKRTDGDADYDEYEEDGTTPTPDPTAPTAKPRLRGNKP
54	DP00342	MSGGDGRGHNTGAHSTSGNINGGPTGIGVSGGASDGSGWSSENNPWGGGSGSGIHWGGGSG
55	DP01052	PAPPVLPSYIDPAKKSFAARMMEKMGWKHGEGLGVNKQGITAPLVAKKTAMSSGVIVQGAE
56	DP00448	SDEIMDLLVQSVTKSSPRALAARERKRSRGNRKSLRRTLKSGLGDDLVQALGLSKGPGLEV
57	DP00719	QEPVQEAPKGRKRKPRTTEPKQPVEPKKPVESKKSGKSAKSKEKQEKITDTFKVKRKVDRF
58	DP00672	RRSRRVKANDRERNRMHNLNAALDALRSVLPSFPDDTKLTKIETLRFAYNYIWALAETLRL
59	DP00824	APQYAPGDEPSYDEDTDDSDKLVENDTSITDEDYAAIEASLSETFNTAADPGRRLGEGSKP
60	DP00123	ALIVLGGVAGLLLFIGLGIFFCVRCRHRRRQAERMSQIKRLLSEKKTCQCPHRFQKTCSPI
61	DP00977	YPERDSAKDGNQEKERALLVKVQERYQGNQGDYDEYDQDETTPPPDPTAQTARPRLRQNQD
62	DP00835	FRASVSPSSLPRASAFLSQNESSSKLSNASNSPGMLANSSTATSASANERLQEKQTESLSN
63	DP00014	TVTTHSSDSEVPSRVTEVVVKLFDSDPITVVLPEEVSKDNPKFMDTVAEKALQEYRRKSRME
64	DP00278	SPSKDCGSPKYAYFNGCSSPTAPLSPMSPPGYKLVTGDRNNSSCRNYNKQASEQNWANYSAE
65	DP00389	DALAYGSQGNLNPLINEISKIISAAGNFDVKEERAAASLLQLSGNASDFSYGRNSITLTASA
66	DP00592	MSERQGAGATNGKDKTSGENDGQKKVQEEFDIDMDAPETERAAVAIQSQFRKFQKKKAGSQS
67	DP00057	MARYRRSRTRSRSPRSRRRRRRSGRRRSPRRRRRYGSARRSRRSVGGRRRRYGSRRRRRRY
68	DP00090	MLAARTGAAGSQISEENTKLRRQSGFSVAGKDKSPKKASENAKDSSLSPSGESQLRARQLAL
69	DP00812	MIGKNIKSLRKTHDLTQLEFARIVGISRNSLSRYENGTSSVSTELIDIICQKFNVSYVDIVGE
70	DP00543	MPGLVDSNPAPPESQEKKPLKPCCACPETKKARDACIIEKGEEHCGHLIEAHKECMRALGFKI
71	DP00152	QEREKDIKQEGETAICSEMTADRDPVYDESTDEESENQTDLSGLASAAVSKDDSIISSLDVTD
72	DP00220	RYLHTALEGMANPEDPECESEGWLLEKSVPETWKAFLESVKKLGKGNQVEAEGEDAGQAPAAG
73	DP00718	SPPYYSEKTQLYNKPHEEPSNSLMAIECRVCGDKASGFHYGVHACEGCKGFFRRTIRLKLIYD
74	DP00709	MPFFGNTFSPKKTPPRKSASLSNLHSLDRSTREVELGLEYGSPTMNLAGQSLKFENGQWIAET
75	DP01084	LGCPEKMGDPQPLGPRSAEPQQNPNLGSTGFYGVKSEPTQDTKPQFPRQMPSRNASGGQGSST
76	DP00378	PNLQNQQVLTGLPGVMPNIQYQVIPQFQTVDGQQLQFAATGAQVQQDGSGQIQIIPGANQQII
77	DP00064	MSGLFHHRTKPREIRAFVMATRLTKKQLAQAIQNTLPNPPRRKRRAKRRAAQVPKPTQAGVSMA
78	DP00877	MAQEQTKRGGGGGDDDDIAGSTAAGQERREKLTEETDDLLDEIDDVLEENAEDFVRAYVQKGGQ
79	DP00462	MLSVRTPLATIADQQQLQLSPLKRLTLADKENTPPTLSSTRVLASKAARRIFQDSAELESKAPT
80	DP00656	MTAAQAKTTKKNTAAAAQEAAGAAQPSGLGLDSIGDLSSLLDAPAASQGGSGPIELDLDLIDED
81	DP01038	MADNKQSFQAGQAAGRAEEKGNVLMDKVKDAATAAGASAQTAGQKITEAAGGAVNLVKEKTGMNK
82	DP00026	MAQVINTNSLSLLTQNNLNKSQSALGTAIERLSSGLRINSAKDDAAGQAIANRFTANIKGLTQAS
83	DP01011	VEVDCSRFPNTTNEEGKDVLVCTEDLRPICGTDGVTHSECLLCAYNIEYGTNISKEHDGECREAV

84	DP00008	MLWQKSTAPEQAPAPPRPYQGVRVKEPVKELLRRKRGHTSVGAAGPPTAVVLPHQPLATYSTVGP
85	DP00678	TTQITKTVKGGISETRIEKRIVITGDADIDHDQVLVQAIKEAKEQHPDMSVTKVVVHQETEIADE
86	DP00722	LGGRQGGGAPAGGNIGGGQPQGGWGQPQQPQGGNQFSGGAQSRPQQSAPAAPSNEPPMDFDDDIP
87	DP01034	MSTQQQARALMMRHHQFIKNRQQSMLSRAAAEIGVEAEKDFWTTVQGKPQSSFRTTYDRSNASLS
88	DP00018	KPSACRNLFGPVDHEELTRDLEKHCRDMEEASQRKWNFDFQNHKPLEGKYEWQEVEKGSLPEFYYR
89	DP00468	MFQAAERPQEWAMEGPRDGLKKERLLDDRHDSGLDSMKDEEYEQMVKELQEIRLEPQEVPRGSEPW
90	DP00948	QASSRSSSRSRGNSRNSTPGSSRGNSPARMASGGGETALALLLLDRLNQLESKVSGKGQQQQGQTV
91	DP01054	MMCGAPSATQPATAETQHIADQVRSQLEEKENKKFPVFKAVSFKSQVVAGTNYFIKVHVGDEDFVHL
92	DP00157	PVQATVHGVDDNKQQSQTAEIVNVKPLASQLAGVENVKTDTLQSDTTVITGNKAGTTDNDNSQTDKT
93	DP00978	TLNCTDLRNATNTTSSSWETMEKGEIKNCSFNITTSIRDKVQKEYALFYNLDVVPIDNASYRLISCN
94	DP00788	VLDGNKNQFEPSISRSNFQGKHTKFENDELAESTTTKIIDSTDHIRKASSKKSKRLDKVGKKKGGKK
95	DP01105	ENREIQPPFKPKVCGKGAENFDKFFTRGQPVLTPPDQLVIANIDQSDFEGFSYVNPQFVHPILQSAV
96	DP00081	EGSIGNGLDLTGILFGNIDSEGRLLQDDDGEGRGGTGFDAELRENIGSLSKLGLDSMLLEVIDLKEA
97	DP00747	EVVPPQVLSEPNEEAGAALSPLPEVPPWTGEVSPAQRDGGALGRGPWDSSDRSALLKSKLRALLTAPR
98	DP00179	MNTDQQKVSEIFQSSKEKLQGDAKVVSDAFKKMASQDKDGKTTDADESEKHNYQEQYNKLKGAGHKKE
99	DP00328	DPNNMIPVTSANAATGEITPAQGATGSSGGNTSGVLSTPKAKRAKHPPGTEKPRSRSQSEQPATCPIC
100	DP00753	EVDSTEDIKKAFEIFDKEKNGYISASELKHVLTTLGEKLTEQEVDDLLKEIGVEEGLINVDDFVKLITS
101	DP00834	GTKKLGSIGSDLGASIKGFKKAMSDDEPKQDKTSQDADFTAKTIADKQADTNQEQAKTEDAKRHDKEQV
102	DP01002	MKKFLPGLLLMGLVACSSNQRMSDYSQPESQSDLAPKSSTTQFQPQPLLSKASSMPLNLLSSSKNGQVSP
103	DP00976	VSLKCTDLKNDTNTNSSSGRMIMEKGEIKNCSFNISTSIRGKVQKEYAFFYKLDIIPIDNDTTSYKLTSC
104	DP00345	GNGKITQDELSKVVDNYELLKHSKNVTNSLDKLISSVSAFTSSNDSRNVLVAPTSMLDQSLSSLQFARAA
105	DP00539	PQAIPKKRGRKPGSVVAAAAAEAKKKAVKESSIRSVQETVLPIKKRKTRETVSIEVKEVVKPLLVSTLGE
106	DP00042	MPKRKSATKGDEPARRSARLSARPVPKPAAKPKKAAAPKKAVKGKKAAENGDAKAEAKVQAAGDGAGNAK
107	DP00048	MGGKWSKSSVVGWPTVRERMRRAEPAADGVGAASRDLEKHGAITSSNTAATNAACAWLEAQEEEEVGFPV
108	DP00527	MKSTSLFLCSLLLLLVTGAIGRKTKEKYSQSEEVVSESFASGPSSGSSDDELVRDKPYGPKVSGGSFGEE
109	DP00752	MQVQVKTLEGETKIYTLEQGTSVLDLKSQISQDMGFEIDMMTLVNNGFIAPNTELVTDDVTYYLSLKLLGG
110	DP00333	DAKKPEDWDEEMDGEWEPPVIQNPEYKGEWKPRQIDNPDYKGTWIHPEIDNPEYSPDPSIYAYDNFGVLGL
111	DP00017	TSACRSLFGPVDHEELSRELQARLAELNAEDQNRWDYDFQQDMPLRGPGRLQWTEVDSDSVPAFYRETVQV
112	DP00771	MSDYEEAFNDGNENFEDFDVEHFSDEETYEEKPQFKDGETTDANGKTIVTGGNGPEDFQQHEQIRRKTLKE
113	DP00841	LNLDISPQRPRPTLQLPLANDGGSRSPSSESSPQHPTPPARPRHMLGLPSTLFTPRSMESIEIDQKLQEIM
114	DP00719	AAYGGAYGENPCSSEPCGFSSNGLIESVELRGESAFSGIPNGQWMTQSFTDQIPSFSNHCGTQEQEEESHA
115	DP00636	MAGPQRATSGLPTRRTTTRQPTRRAGSAIPERQTSTASPAVSTKTAAISRTRTLKSPGEPASVLAKRKERD
116	DP00343	EQVSHGTQNRPLLRNSLDDLVGPPSNLEGQSDERALLDQLHTLLSNTDATGLEEIDRALGIPELVNQGQAL
117	DP00608	GDGGAFPEIHVAQYPLDMGRKKKMSNALAIQVDSEGKIKYDAIARQGQSKDKVIYSKYTDLVPKEVMNADD
118	DP00669	GDIPDPGSKPTPPGMADELPTETYDLPPEIYTTTFLPRTIYPQEEMPYDDKPFPSLLSKANDLNAVFEGPA

119	DP00862	AVQMQSEKSASDSGEVATEKAPAKQDTKEKSGTETEKGKEDGTKGTKDSSADKETSAEASEKGTVVTETAD
120	DP00702	PKSHSRQASTDAGTAGALTPQHVRAHSSPASLQLGAVSPGTLTPTGVVSGPAATPTAQHLRQSSFEIPDDV
121	DP00435	FEEAMRFARRSVSDNDIRKYEMFAQTLQQSRGFGSFRFPSGNQGGAGPSQGSGGGTGGSVYTEDNDDDLYG
122	DP00094	MPTPSAPSPQPKGFRRAVSEQDAKQAEAVTSPRFIGRRQSLIEDARKEREAAAAAAAAAVASSEPGNPLEA
123	DP00768	KISEGWRDEPTDAALGPEPPGPALEIKPMLQAQESPSLPAPGPPEPEEEPIPEPQPTIQASSLPPPQDSARL
124	DP00853	MGMLELVGEYWEQLKITVVPVVAAAEDDDNEQHEEKAAEGEEKEEENGDEDEDEDEDEDDDDDDDEDEEEEE
125	DP00863	TLREKLMNFLVRHMNEKRELTLPVTLEELSRLFGCARPALSRVFQELEREGYIEKHGRRIKVLKNPFEHDRI
126	DP00055	HHSGSMDQRQKLQHWIHSCLRKADKNKDNKMNFKELKDFLKELNIQVDDGYARKIFRECDHSQTDSLEDEEIE
127	DP00806	VTADVVSAQALTDEQKARLADTLAAKSGKTVKLNARVDESLIGGMIVKLGSQMIDSSIRSKLASLQNAMKEVG
128	DP00159	FIDENLKAADSDPTAPPYDSLLVFDYEGSGSEAASLSSLNSSESDQDQDYDYLNEWGNRFKKLADMYGGGEDD
129	DP00086	MEEPQSDPSVEPPLSQETFSDLWKLLPENNVLSPLPSQAMDDLMLSPDDIEQWFTEDPGPDEAPRMPEAAPPV
130	DP00815	YHPADKDYGLMKIDEPNTPYHNMIGDDEDAYSDSEGNEVMTPDILAKKLAAAEGSEPKYRTREQESSGEEDND
131	DP00086	KKPLDGEYFTLQIRGRERFEMFRELNEALELKDAQAGKEPGGSRAHSSHLKSKKGQSTSRHKKLMFKTEGPDSD
132	DP00707	GVDPALSDPVLARRLRENRSLAMSRLDEVISKYAMLQDKSEEGERKKRRARLQGTSSHSADTPEASLDSGEGPS
133	DP00146	MARYFRRRKFCRFTAEGVQEIDYKDIATLKNYITESGKIVPSRITGTRAKYQRQLARAIKRARYLSLLPYTDRHQ
134	DP00490	QSVSPMRSVSENSLVAMDFSGQKTRVIDNPTEALSVAVEEGLAWRKKGCLRLGNHGSPTAPSQSSAVNMALHRSQ
135	DP00539	MVAGMLGLREEKSEDQDLQGLKDKPLKFKKVKKDKKEEKEGKHEPVQPSAHHSAEPAEAGKAETSEGSGSAPAVP
136	DP00919	MGGKWSKSSIVGWPAIRERLRRAQPAAERRRAQPAAEGVGAASQDLGRHGALTSSNTDTTNADEEEEVGFPVRPQ
137	DP00201	NKELDPVQKLFVDKIREYRTKRQTSGGPVDAGPEYQQDLDRELFKLKQMYGKADMNTFPNFTFEDPKFEVVEKPQS
138	DP00016	RQNPCGSKACRRLFGPVDSEQLSRDCDALMAGCIQEARERWNFDFVTETPLEGDFAWERVRGLGLPKLYLPTGPRR
139	DP00932	MKTIEQKIEQCRKWQKAARERAIARQREKLADPVWRESQYQKMRDTLDRRIAKQKERPPASKTRKSAVKIKSRGLK
140	DP01086	LEVVFKALDSEIKCEAEKQEEKPAILLSPKEESVVLSKPTNAPPLPPVVLKPKQEVKSASQIVNEQRGNAAPAARL
141	DP00015	MTDVETTYADFIASGRTGRRNAIHDILVSSASGNSNELALKLAGLDINKTEGEEDAQRSSTEQSGEAQGEAAKSES
142	DP00970	MDIRKIKKLIELVEESGISELEISEGEESVRISRAAPAASFPVMQQAYAAPMMQQPAQSNAAAPATVPSMEAPAAAE
143	DP00075	PPKTFEKSMMNLQTKIDAKKEQLADARRDLKSAKADAKVMKDAKTKKVVESKKKAVQRLEEQLMKLEVQATDREENK
144	DP00551	MDPQTAPSRALLLLLFLHLAFLGGRSHPLGSPGSASDLETSGLQEQRNHLQGKLSELQVEQTSLEPLQESPRPTGVW
145	DP00990	MSFTIPTNLYKPLATKPKHLSSSSFAPRSKIVCQQENDQQQPKKLELAKVGANAAAALALSSVLLSSWSVAPDAAMA
146	DP00797	LPIFTPAKMGATKEEREDTPIQLQELLALETALGGQCVDRQEVAEITKQLPPVVPVSKPGALRRSLSRSMSQEAQRG
147	DP00328	AKPGVKKEKKTTSGKKSSSGSSGSGSGALSSSGSVPQVQTVQSLHTLQGVQVKKDPDAQQQQQQQQQQQQQQQAMT
148	DP00469	MQEQATSSLAASSLPSSSERSSSSAPHLEIKEGIESDEEIRRVPEFGGEAVGKETSGRESGSATGQERTQATVGESQ
149	DP00534	AAPEGGISDVVEKSIKEAQETCAGDPVSGECVAAWDEVEELSAAASHARDKKKADGSDPLEEYCKDNPETNECRTYDN
150	DP00223	MALRVTRNSKINAENKAKINMAGAKRVPTAPAATSKPGLRPRTALGDIGNKVSEQLQAKMPMKKEAKPSATGKVIDKK
151	DP00943	DEEDGEPPYEPESGCVEIPGLSEEEDPAPSRKIHFSTAPIQVFSTYSNEDYDRRNEDVDPMAASAEYELEKRVERLEL
152	DP00447	DGIRDAMVGLREEMIEKIRTEALMTNDRLEAMARLRNEESEKMAKDTSDEVSLNPTSEKLNNLLEGNDSDNDLSLEDF
153	DP00087	TAPITDVSLGDELRLDGEEVDMTPADALDDFDLEMLGDVESPSPGMTHDPVSYGALDVDDFEFEQMFTDAMGIDDFGG

154	DP01083	VKSAGEVGVKIGNPVPYNEGHAQQQAVSAPASAATPPASKPQPQNGSLGVGSTVAKAYGASKPFGKPAGTGLLQPTSGT
155	DP00144	EVKFERLVKEEKLKTPYKRRHSFTPPSNENSRSNSPNSSNSSANGDAAAPKGGNNPHSRNSKKSGNFRAHKEEKRVRHN
156	DP01070	SDKQIKNGECDKAYLDELVELHRRLMTLRERHILQQIVNLIEETGHFHITNTTFDFDLCSLDKTTVRKLQSYLETSGTS
157	DP00061	LKSAEAVGVKIGNPVPYNEGLGQPQVAPPAPAASPAASSRPQPQNGSSGMGSTVSKAYGASKTFGKAAGPSLSHTSGGT
158	DP00420	MLENVQQELVGEPRPQAPPSLPTQGPSCPAEDGPPALKEKEEPHYIEIWKKHLEAYSGCTLELEESLEASTSQMMNLNL
159	DP00854	SGQGRGGQGGYGGGGGGQGGGGWGGGPGGGQQGGGAPADDPWATGGAPAGGQQGGGGQGGGGWGGGSGGGGGYSDEPPF
160	DP00994	NVKSKQLKTKEYLEFDATFVESNTRKNFGDRNNNSRNNFNNRRGGRGARKGNNTANATNSANTVQKNRNIDVSNLPSLA
161	DP00242	KIRTLQGRVVSDKMEKSIVVAIERFVKHPIYGKFIKRTTKLHVHDENNECGIGDVVEIRECRPLSKTKSWTLVRVVEKAV
162	DP00359	SGQPAVDLNKKVQDAVKEAEDACAKGTSADCAVAWDTVEELSAAVSHKKDAVKADVTLTDPLEAFCKDAPDADECRVYED
163	DP00851	HGFSLAFLIVAIGPFMIIPNVGLNEWGHTFWFMEELFVAPLHWGFVFFGWMALGVFGVVLQILMGVKRLIGKDCVAALVG
164	DP00753	MSEQKKVLTAEEQQEYKEAFQLFDKDNDNKLTAEELGTVMRALGANPTKQKISEIVKDYDKDNSGKFDQETFLTIMLEYGQ
165	DP00020	SAAKRARNYSSQRNVQPTANAASANAAAAAAAAASNSYAIPENELLDEDTMNFISSLKNDLSNISNSLPFEYPHEIAEAIR
166	DP00775	EGIRDDRNEEKRITEAEKNERVQRQLLTLSSELSQARDENKRTHNDIIHNENMRQGRDKYKTLRQIRQGNTKQRIDEFEAL
167	DP00217	MEEDYSWAEEEDEGEAEGEEEEEEEEDQESPPKAVKRPAATKKAGQAKKKKLDKEDESSEEDSPTKKGKGAGRGRKPAAKK
168	DP00447	SARDGIRDAMVGLREEMIEKIRTEALMTNDRLEAMARLRNEESEKMAKDTSDEVSLNPTSEKLNNLLEGNDSDNDLSLEDF
169	DP00720	DVDFGDSDNENEPDAYLARLKAEAREKEEDDDDGDSDEESTDEDFKPNENESDVAEEYDSNVESDSDDDSDASGGGGDSDGA
170	DP00463	GGAQTPNATSEGCQIIHPPWEGGIRYRGLTRDQVKAINFLPVDYEIEYVCRGEREVVGPKVRKCLANGSWTDMDTPSRCVRI
171	DP00588	MSTNPKPQRKTKRNTNRRPQDVKFPGGGQIVGGVYLLPRRGPRLGVRATRKTSERSQPRGRRQPIPKARRPEGRTWAQPGYP
172	DP00937	GGEDDELKGLLGLPEEETELDNLTEFNTAHNKRISTLTIEEGNLDIQRPKRKRKNSRVTFSEDDEIINPEDVDPSVGRFRNM
173	DP00510	MATFPPATSAPQQPPGPEDEDSSLDESDLYSLAHSYLGGGGRKGRTKREAAANTNRPSPGGHERKLVTKLQNSERKKRGARR
174	DP00156	TKAGQGTSAPPEASPTAAPESSTSFPPAPTSGMSHPPPAAREDKSPSEESAPTTSPESVSGSVPSSGSSGREEDAASTLVTG
175	DP00242	MTDKIRTLQGRVVSDKMEKSIVVAIERFVKHPIYGKFIKRTTKLHVHDENNECGIGDVVEIRECRPLSKTKSWTLVRVVEKAV
176	DP00342	MSGGDGRGHNTGAHSTSGNINGGPTGIGVSGGASDGSGWSSENNPWGGGSGSGIHWGGGSGRGNGGGNGNSGGGSGTGGNLSA
177	DP00980	SGKAVAKEDRTTTPYMTKYERARILGTRALQISMNAPVLVDLEGETDPLQIAMKELAQKKIPLLVRRYLPDGSYEDWSVAELI
178	DP00229	LNKWKSKGRRFKGKGKGNKAAQPGSGKGKVQFQGKKTKFASDDEHDEHDENGATGPVKRAREETDKEEPASKQQKTENGAGDQ
179	DP00048	MGGKWSKSSVVGWPTVRERMRRAEPAADGVGAASRDLEKHGAITSSNTAATNAACAWLEAQEEEEVGFPVTPQVPLRPMTYKA
180	DP00177	PEEQEEEPQPRKPGTRRERTLGAPASSERSAAGGRGPRGHKRKLNEEDAASESSRESSNEDEGSSSEADEMAKALEAELNDLM
181	DP00798	MENKDALDIGKDDTNTSEADVSKNETQEQPVLSKSALKRLKRQQEWDAGREKRAEMRREKKRLRKEERKRKIEAGEVVKSQKK
182	DP01092	IELSRLIGLLLETEKRKNTEQKESGTNKIEDKVTLSKIAQELSKNDVEEKDLEKKVKELKEKIEKGEYEVSDEKVVKGLIEFFT
183	DP00091	MEPEPEPEQEANKEEEKILSAAVRAKIERNRQRALMLRQARLACRPYPTGEGISTVKAPPKVIDSGGGFFIEEEEAEEQHVENV
184	DP00306	MSSENCFVAENSSLHPESGQENDATSPHFSTRHEGSFQVPVLCAVMNVVFITILIIALIALSVGQYNCPGQYTFSMPSDSHVSS
185	DP00187	KKRPKPGGWNTGGSRYPGQGSPGGNRYPPQGGGTWGQPHGGGWGQPHGGGWGQPHGGGWGQPHGGGWGQGGGTHNQWNKPSKPK
186	DP00053	MLEEALAAIQNARDLEELKALKARYLGKKGLLTQEMKGLSALPLEERRKRGQELNAIKAALEAALEAREKALEEAALKEALERE
187	DP00140	MAHKKAGGSTRNGRDSEAKRLGVKRFGGESVLAGSIIVRQRGTKFHAGANVGCGRDHTLFAKADGKVKFEVKGPKNRKFISIEAE
188	DP00091	IKRSLEVWGSEEALEEAKEVRKDNRDKMKQKKFDKKVKELRRTVRSSLWKKEASGHQHEYGPEEHVEEDSYKKTCITCGYEMNYE

189	DP01074	MPPIATRRGQYEPKVQQAKLSPDTIPLNPADKTKDPLARADSLHHHVESDSQEDDKAAEEPPLSRKRWQNRTFRRKGRRQAPYKHK
190	DP00499	LTAATHWASMDPAVVHPELNGAAYSRYPPGVVSVAPTGIPAAVEGIVPSAMSLSHGLPPVAHPPHAPSPGQTVKPEADRDHTTDQL
191	DP00768	GTPQKTKQEEEGTEPEPELEPKPETAPEETELEEVSLPPEEPCVGKEVAAVTLGPQGTQETALTPPTSLQAQVSVAPEAHSSPRGW
192	DP00032	ADPMVDNRITKKRYRLDHLLKEQPEEVLKRLYRAMEVEEENLHALVQAMMQAPERAGGAMTAAGVLDPASGEPGDWTPPRYFNMMF
193	DP00929	MEPVDPRLEPWKHPGSQPKTACTNCYCKKCCFHCQVCFITKALGISYGRKKRRQRRRAHQNSQTHQASLSKQPTSQPRGDPTGPKE
194	DP00869	AQPKSGFSTDRPLWYPGAKAPEYLDGSLVGDYGFDPFGLGKPAEYLQYDYDGLDQNLAKNLAGDIIGTRTESADVKSTSLQPYSEVF
195	DP00423	TVTAKKASSPSSLTYKEMILKSMPQLNDGKGSSRIVLKKYVKDTFSSKLKTSSNFDYLFNSAIKKCVENGELVQPKGPSGIIKLNKK
196	DP00347	MNLEPPKAEIRSATRVMGGPVTPRKGPPKFKQRQTRQFKSKPPKKGVQGFGDDIPGMEGLGTDITVICPWEAFNHLELHELAQYGII
197	DP00157	TGETTSFDEVDGVTSKSIIGKPVQATVHGVDDNKQQSQTAEIVNVKPLASQLAGVENVKTDTLQSDTTVITGNKAGTTDNDNSQTDKT
198	DP00423	KASSPSSLTYKEMILKSMPQLNDGKGSSRIVLKKYVKDTFSSKLKTSSNFDYLFNSAIKKCVENGELVQPKGPSGIIKLNKKKVKLST
199	DP00260	MPLNVSFTNRNYDLDYDSVQPYFYCDEEENFYQQQQQSELQPPAPSEDIWKKFELLPTPPLSPSRRSGLCSPSYVAVTPFSLRGDNDG
200	DP01085	ETIGNPTIFGETDTEAQKTFSGTGNIPPPNRVVFNEPMVQHSVNRAPPRGVNIQNQANNTPSFRPSVQPSYQPPASYRNHGPIMKNEA
201	DP01014	MSTDVAAAQAQSKIDLTKKKNEEINKKSLEEDDEFEDFPIDTWANGETIKSNAVTQTNIWEENWDDVEVDDDFTNELKAELDRYKRENQ
202	DP00461	GSNGADNAHNNAFGGGKNPGIGNTSGAGSNGSASSNRGNSNGWSWSNKPHKNDGFHSDGSYHITFHGDNNSKPKPGGNSGNRGNNGDGA
203	DP00136	AKRKASGPPVSELITKAVAASKERSGVSLAALKKALAAAGYDVEKNNSRIKLGLKSLVSKGTLVQTKGTGASGSFKLNKKAASGEAKPK
204	DP00195	MPKRKAEGDAEGDKAKVKDEPQRRSARLSAKPAPPKPEPKPKKAPAKKGEKVPKGKKGKADAGKDGNNPAENGDAKTDQAQKAEGAGDA
205	DP01088	MAKSKEDITYATSQARLSEDEAVRVAYEHGSPLEGGKIADSQPVDLFSSAHNMPKSGQTTMDSNTSDQSQMQRDTQEGGSKEFTTGAPG
206	DP00985	EKNGTLIMIDDERILEHLTDEEKSRIVKKKVRFRTLGCYPLTGAVESEATSLTDIIQEMLLTRTSERQGRVIDHDGAGSMEEKKRQGYF
207	DP00141	ALAPDSPDNDLRAGQFGISARKPFTTLGEVAPVWVPDSQAPNCMKCEARFTFTKRRHHCRACGKVFCASCCSLKCKLLYMDRKEARVCV
208	DP00461	MGSNGADNAHNNAFGGGKNPGIGNTSGAGSNGSASSNRGNSNGWSWSNKPHKNDGFHSDGSYHITFHGDNNSKPKPGGNSGNRGNNGDGA
209	DP00997	QDEADELTRRFVALMDAGEPADSEGAMDAAEDHRQGIARNHYDCGYEMHTCLGEMYVSDERFTRNIDAAKPGLAAYMRDAILANAVRHTP
210	DP00039	MPKRKAEGDAKGDKAKVKDEPQRRSARLSAKPAPPKPEPKPKKAPAKKGEKVPKGKKGKADAGKEGNNPAENGDAKTDQAQKAEGAGDAK
211	DP00191	CLKGTGENYRGNVAVTVSGHTCQHWSAQTPHTHNRTPENFPCKNLDENYCRNPDGKRAPWCHTTNSQVRWEYCKIPSCDSSPVSTEQLAP
212	DP00631	MTPSTPPRSRGTRYLAQPSGNTSSSALMQGQKTPQKPSQNLVPVTPSTTKSFKNAPLLAPPNSNMGMTSPFNGLTSPQRSPFPKSSVKRT
213	DP00864	NKAVQNDSNPSASQPTTGPSAASPASENQNGNGLSAPPGPGGGPHPPHTPSHPPSTRITRSQPNHTPAGPPGPSSNPVSNGKETRRSSKR
214	DP00486	PEKVGPKQAEDAAKSITNGSDDGAQPSTSTAQEQDDVLIVDSDEEDSSNNADVSEEERSRKRKLDEKENLSAKRSRIEQKEELDDVIALD
215	DP00353	MMNNNGNQVSNLSNALRQVNIGNRNSNTTTDQSNINFEFSTGVNNNNNNNSSSNNNNVQNNNSGRNGSQNNDNENNIKNTLEQHRQQQQA
216	DP00675	DTKEEGATKKKTQKPDRLERGKMKIVPKESEKDSKTKPPDATIVVEGVKYQVRKKGKTKSKNTQDGLYHNKNKPQESRKKLEKALLAWAII
217	DP01042	YANEPKVCFYMDDDYNGESLCAAQGNSVASIPDKWNDRISSISIPHGLVVTVYEDVDFLGASRSFEADVDLVSDKDLIYLNDNISAFKIKKA
218	DP00902	PTSIHETVYENDGDPITLLDQIADADEASWFDKIALKKAIEELDERERLIVYLRYYRDRTQSEVASRLGISQVQVSRLEKKILQHIKDKMDG
219	DP01033	MSYRKELEKYRDLDEDKILGALTEEELRKLENELEELDPDNALLPAGLRQRDQTQKPPTGPFKREELMAHLEQQAKDIKDREDLVPFTGEKR
220	DP00086	MEEPQSDPSVEPPLSQETFSDLWKLLPENNVLSPLPSQAMDDLMLSPDDIEQWFTEDPGPDEAPRMPEAAPPVAPAPAAPTPAAPAPAPSWPL
221	DP00808	GLPTTRPIAPMATGVLGQHTPVTLDLPPPADTQQKPVLPGPTAVVVTRPSRASLRRAASGPRSLRPVASGNWQSTLNSIVGLGVQSLKRRRCF
222	DP00113	MSSSTPFDPYALSEHDEERPQNVQSKSRTAELQAEIDDTVGIMRDNINKVAERGERLTSIEDKADNLAVSAQGFKRGANRVRKAMWYKDLKMK
223	DP00944	MADNEEDVEAEEEYTELTDISGVGPSKAESLREAGFESVEDVRGADQSALADVSGIGNALAARIKADVGGLEVESETEAEVEEEGGEEAPDEDV

224	DP00016	MSEPAGDVRQNPCGSKACRRLFGPVDSEQLSRDCDALMAGCIQEARERWNFDFVTETPLEGDFAWERVRGLGLPKLYLPTGPRRGRDELGGGRR
225	DP00018	ESQDVSGSRPAAPLIGAPANSEDTHLVDPKTDPSDSQTGLAEQCAGIRKRPATDDSSTQNKRANRTEENVSDGSPNAGSVEQTPKKPGLRRRQT
226	DP00615	SPDADLIEANLLWRQEMGGNITRVESENKVVILDSFEPLQAEEDEREVSVPAEILRRSRKFPRAMPIWARPDYNPPLLESWKDPDYVPPVVHGC
227	DP01031	NTYDVDMVDANLLMEGGVAQTEPESRVPVLDFLEPMAEEESDLEPSIPSECMLPRSGFPRALPAWARPDYNPPLVESWRRPDYQPPTVAGCALP
228	DP00092	SDDELGSEEDGFDGATAAARKEVIRNKIRAIGKMARVFSVLREESESVLTLKGLTPTGMLPSGVLSGGKQTLQSATVEAIEADEAIKGFSPQHK
229	DP00984	ELSPSLVISLSTGLSLFLGRFVFFNFQRENMAKQVPEQNGMSHFEAGDTRAKEYVSLLKSNDPVGFNIVDVLAWGSIGHIVAYYILATASNGYDP
230	DP00984	PSLVISLSTGLSLFLGRFVFFNFQRENMAKQVPEQNGMSHFEAGDTRAKEYVSLLKSNDPVGFNIVDVLAWGSIGHIVAYYILATASNGYDPSFF
231	DP00092	DDELGSEEDGFDGATAAARKEVIRNKIRAIGKMARVFSVLREESESVLTLKGLTPTGMLPSGVLSGGKQTLQSATVEAIEADEAIKGFSPQHKIT
232	DP00552	YNGQSETSQQPETPVFNTLPMMGKASPVSLGVPSEATANNGQQQQVQEQRRRINAMLQDYELQRRLHSEQLQFEQAQTQQAAVQVPGIQTLGTQSQ
233	DP00615	ATCTTRHDSPDADLIEANLLWRQEMGGNITRVESENKVVILDSFEPLQAEEDEREVSVPAEILRRSRKFPRAMPIWARPDYNPPLLESWKDPDYVP
234	DP00517	GGGVSPDVCPALSDEPGGLTASRGRVHEASPPTFQKDALLGSKPNKPSLPSSSQNLGQTEVSKVSETVQEELTPPPQKAAPQGKSKSDPLKKKTDR
235	DP00092	SDDELGSEEDGFDGATAAARKEVIRNKIRAIGKMARVFSVLREESESVLTLKGLTPTGMLPSGVLSGGKQTLQSATVEAIEADEAIKGFSPQHKIT
236	DP00491	VVEWTNNTLPLEPCPKEQIIGLSRRIADENGKPRPAFSNALEPDFKALSIAVTGSGSCRHLQFIPVAPPSPGSSAAPATEVPDRDPEKSSEDDVYLH
237	DP00615	RHDSPDADLIEANLLWRQEMGGNITRVESENKVVILDSFEPLQAEEDEREVSVPAEILRRSRKFPRAMPIWARPDYNPPLLESWKDPDYVPPVVHGC
238	DP00994	ERIETAEKEAYVPATKVKNVKSKQLKTKEYLEFDATFVESNTRKNFGDRNNNSRNNFNNRRGGRGARKGNNTANATNSANTVQKNRNIDVSNLPSLA
239	DP00082	LEAEIETTTDETDDGTNTVSHILNVLKDATPIEDVFSFNYPEGIEGPDIKYKKEHVKYTYGPTFLLQFKDKLNVKADAEWVQSTASKIVIPPGMGRGN
240	DP00097	AKGDEPKRSVAFKKTKKEVKKVATPKKAAKPKKAASKAPSKKPKATPVKKAKKKPAATPKKAKKPKVVKVKPVKASKPKKAKTVKPKAKSSAKRASKK
241	DP00136	AKKAGAAKAKKPAGAAKKPKKATGTATPKKSTKKTPKKAKKPAAAAGAKKAKSPKKAKATKAKKAPKSPAKARAVKPKAAKPKTSKPKAAKPKKTAAK
242	DP00265	KKRPKPGGWNTGGSRYPGQGSPGGNRYPPQGGTWGQPHGGGWGQPHGGSWGQPHGGSWGQPHGGGWGQGGGTHNQWNKPSKPKTNLKHVAGAAAAGAV
243	DP00968	MMDLELPPPGLQSQQDMDLIDILWRQDIDLGVSREVFDFSQRQKDYELEKQKKLEKERQEQLQKEQEKAFFAQFQLDEETGEFLPIQPAQHIQTDTSG
244	DP00024	MHGDTPTLHEYMLDLQPETTDLYCYEQLNDSSEEEDEIDGPAGQAEPDRAHYNIVTFCCKCDSTLRLCVQSTHVDIRTLEDLLMGTLGIVCPICSQKP
245	DP00720	GGDSDNEKGGKSSKKRKTEPSPSKKANTSGSGFKSKEYISDDDSTSSDDEKDNEPAKKKSKPPSDGDAKKKKAKSESEPEESEEDSNASDEDEEDEASD
246	DP00783	LCKKRPKPGGGWNTGGSRYPGQGSPGGNRYPPQGGGGWGQPHGGGWGQPHGGGWGQPHGGGWGQPHGGGWGQPHGGGGWGQGGTHGQWNKPSKPKTNMK
247	DP01057	SQRKKSLIYAAKGDGNILDDLNEATKKASDFVTDKTKEALADGEKAKDYVVEKNSETADTLGKEAEKAAAYVEEKGKEAANKAAEFAEGKAGEAKDATK
248	DP01058	VAQRKKSLIYAVKSDGNILDDLNEATKKASDFVTDKTKEALADGEKTKDYIVEKTIEANETATEEAKKALDYVTEKGKEAGNKAAEFVEGKAEEAKNAT
249	DP00953	MAESPTEEAATAGAGAAGPGASSVAGVVGVSGSGGGFGPPFLPDVWAAAAAAGGAGGPGSGLAPLPGLPPSAAAHGAALLSHWDPTLSSDWDGERTAPQ
250	DP00161	PSPQHQYQPPYASAQPRQPVQQPPEAQVPPQHAPHPAQPVQQPAYQPQPEQPLQQPVSPQVAPAPQPVHSAPQPAQQAFQPAEPVAAPQPEPVAEPAPVM
251	DP00876	MNNQRKKARNTPFNMLKRERNRVSTVQQLTKRFSLGMLQGRGPLKLFMALVAFLRFLTIPPTAGILKRWGTIKKSKAINVLRGFRKEIGRMLNILNRRRR
252	DP00699	MDKLELVNDGLNIIDFIQKNQKEIQKTYGRSSIQQPSIKDQTKAWEDFLQCTSGESEQVEGGMSKDDGDVERRNLEDLSSTSPTDGTIGKRVSNTRDWAE
253	DP01069	MKRLLVLSILLTLGFSFAGTASADENANRPVNPGEDPNEAFRSTPFEATTSALGDCRECIAYRTGATTGKGSRRHDDTVSREIKGSSATPGGSEKAGTGRQ
254	DP00053	MLEEALAAIQNARDLEELKALKARYLGKKGLLTQEMKGLSALPLEERRKRGQELNAIKAALEAALEAREKALEEAALKEALERERVDVSLPGASLFSGGLH
255	DP01003	MEPVDPNLEPWNHPGSQPKTACNNCYCKRCSYHCLVCFQTKGLGISYGRKKRRQRRSAPPSSEDHQNPIPKQPLPQTRGDQTGSEESKKKVESKTETDPFD
256	DP00902	MDVDVKQGQSPIKDQEMKELIRRSQEGDQEARDEIIEKNMRLVWSVVQRFLNRGYEADDLFQIGCIGLLKSVDKFDLSYDVKFSTYAVPMIIGEIQRFLRD
257	DP00458	LHKDVEEAGALQKNHASVTSAQSAEAAEAAETSCLPTAVEPLSMGSCEIPAEQSQCPGPESSPEVNDAEATGKKENSDAKSSTEEPSENVPKVQDNTETPK
258	DP00070	MDVFMKGLSKAKEGVVAAAEKTKQGVAEAAGKTKEGVLYVGSKTKEGVVHGVATVAEKTKEQVTNVGGAVVTGVTAVAQKTVEGAGSIAAATGFVKKDQLGK

259	DP00675	SDTKEEGATKKKTQKPDRLERGKMKIVPKESEKDSKTKPPDATIVVEGVKYQVRKKGKTKSKNTQDGLYHNKNKPQESRKKLEKALLAWAIIAIVLFQVTMG
260	DP00466	KKRPKPGGWNTGGSRYPGQGSPGGNRYPPQGGGGWGQPHGGGWGQPHGGGWGQPHGGGWGQPHGGGWGQGGGTHSQWNKPSKPKTNMKHMAGAAAAGAVVGG
261	DP00895	MEKFAPEFHGEDANNRATKFLESIKGKFTSPKDPKKKDSIISVNSIDIEVTKESPITSNSTIINPTNETDDTAGNKPNYQRKPLVSFKEDPTPSDNPFSKLY
262	DP00864	EPNPEYSTQQAPNKAVQNDSNPSASQPTTGPSAASPASENQNGNGLSAPPGPGGGPHPPHTPSHPPSTRITRSQPNHTPAGPPGPSSNPVSNGKETRRSSKR
263	DP00576	RNISQDMTQTSGTNLTSEELRKRREAYFEKQQQKQQQQQQQQQQGDLSGQSSHPCERPATSSGALGSDLGKACSPFIMFATFTLYLTYELHVIFALHYSSFPL
264	DP00724	QDKKTMMMNEEDDGNGMDELLAVLGYKVRSSEMADVAQKLEQLEVMMSNVQEDDLSQLATETVHYNPAELYTWLDSMLTDLNPPSSNAEYDLKAIPGDAILNQ
265	DP00079	MDIKNSPSSLNSPSSYNCSQSILPLEHGSIYIPSSYVDSHHEYPAMTFYSPAVMNYSIPSNVTNLEGGPGRQTTSPNVLWPTPGHLSPLVVHRQLSHLYAEPQ
266	DP00718	PIDPESDSFTDTLSANISQEMTMVDTEMPFWPTNFGISSVDLSVMEDHSHSFDIKPFTTVDFSSISTPHYEDIPFTRTDPVVADYKYDLKLQEYQSAIKVEPA
267	DP01055	MSSLPFVFGAAASSRVVTAAAAKGTAETKQEKSFVDWLLGKITKEDQFYETDPILRGGDVKSSGSTSGKKGGTTSGKKGTVSIPSKKKNGNGGVFGGLFAKKD
268	DP00473	VNHAETSRLNIERMKQIYQQLSRYRGLCLLASVPSCVEDLSHPVAEPSSSQAGSMSSAGPRPLPSGPASPKRKLEAAEEPPGEELSKRARVAELPTPELPSKDA
269	DP00006	MGDVEKGKKIFVQKCAQCHTVEKGGKHKTGPNLHGLFGRKTGQAPGFTYTDANKNKGITWKEETLMEYLENPKKYIPGTKMIFAGIKKKTEREDLIAYLKKATN
270	DP00673	SKKPGGPGKNRAVNMLKRGMPRGLSLIGLKRAMLSLIDGKGPIRFVLALLAFFRFTAIAPTRAVLDRWRGVNKQTAMKHLLSFKKELGTLTSAINRRSTKQKKR
271	DP00539	VQVKRVLEKSPGKLLVKMPFQTSPGGKAEGGGATTSTQVMVIKRPGRKRKAEADPQAIPKKRGRKPGSVVAAAAAEAKKKAVKESSIRSVQETVLPIKKRKTRE
272	DP00646	PGPPTSLVQSLTPPPDFNQCLKNSSGEKFFSDFSNNMGSRKNPDALATGEVPNQEQIPGEGFIHMHYSQKPEYASGASAGHRLPQGYHSDKRRLSKASSKARSDD
273	DP00661	MSVYFPIHCSDYLRSAEMTEVMMNAPSMEEIGLSPRKDGLSYQIFPDPSDFDRCCKLKDRLPSIVVEPTEGEVESGELRWPPEEFLVQEDEQDNCEETTNEKKDQ
274	DP01036	MASRQNNKQELDERARQGETVVPGGTGGKSLEAQQHLAEGRSKGGQTRKEQLGTEGYQEMGRKGGLSTVEKSGEERAQEEGIGIDESKFRTGNNKNQNQNEDQDK
275	DP00164	MSTESALSYAALILADSEIEISSEKLLTLTNAANVPVENIWADIFAKALDGQNLKDLLVNFSAGAAAPAGVAGGVAGGEAGEAEAEKEEEEAKEESDDDMGFGLFD
276	DP00151	TSHVQEEQIEVEETIEAAKAEEAKDEPPSEGEAEEEGKEKEEAEAEAEAEEEGAQEEEEAAEKEESEEAKEEEGGEGEQGEETKEAEEEEKKDEGAGEEQATKKKD
277	DP00372	MKAKRSHQAVIMSTSLRVSPSIHGYHFDTASRKKAVGNIFENTDQESLERLFRNSGDKKAEERAKIIFAIDQDVEEKTRALMALKKRTKDKLFQFLKLRKYSIKVH
278	DP01042	KKAVCFYGESNFTGDSLCLSSGEQFDLYRGNYPERKQSHLVNPLNDEVYSIKIPPGMQTTVYEDDDYNGKYFVLTEDYTPDDLLIIRMNNKISSMRVSQDEDFICD
279	DP00873	VGQKMTTLQHRSQGDPEDPHDEHYLLATQSKQEQSAKATADRKSASKPIGFEGDLRGQSSDLPERLHSQEVDLGSAQGDGLMAGNDSEEALTALMSRKTAISLFEGK
280	DP00005	MDAQTRRRERRAEKQAQWKAANPLLVGVSAKPVNRPILSLNRKPKSRVESALNPIDLTVLAEYHKQIESNLQRIERKNQRTWYSKPGERGITCSGRQKIKGKSIPLI
281	DP01025	SFAGLKDADVAAALAACSAADSFKHKEFFAKVGLASKSLDDVKKAFYVIDQDKSGFIEEDELKLFLQNFSPSARALTDAETKAFLADGDKDGDGMIGVDEFAAMIKA
282	DP00550	AKDLLKADDIKKALDAVKAEGSFNHKKFFALVGLKAMSANDVKKVFKAIDADASGFIEEEELKFVLKSFAADGRDLTDAETKAFLKAADKDGDGKIGIDEFETLVHEA
283	DP00084	SDNDDIEVESDEEQPRFQSAADKRAHHNALERKRRDHIKDSFHSLRDSVPSLQGEKASRAQILDKATEYIQYMRRKNHTHQQDIDDLKRQNALLEQQVRALEKARSSAQ
284	DP00836	EPYTDSNVPEGDSVPEAHWTKLQHSLDTALRRARSAPTAPIAARVTGQTRNITVDPRLFKKRRLHSPRVLFSTQPPPTSSDTLDLDFQAHGTIPFNRTHRSKRSSTHPVF
285	DP00341	KRLSVELTSSLFRTEPMAWNETADLGLDIGAQGEALGYRQDDPSYRSFHSGGYGQDALGMDPMMEHEMGGHHPGADYPVDGLPDLGHAQDLMDGLPPGDSNQLAWFDTDL
286	DP00655	PSSDYCKKKKKKEKNSKEPVIEDILNLESSSTSSGSPVSPGLYSKTMTPTYDPINGTPALSTMTWFGDSPLTEQNCSVLAFSQPPPSPDVLADMFQPRSLVDKAKMNAGW
287	DP01077	KSNEKKDSGSSKPAFSFGAKPDEKKNDEVSKPAFSFGAKANEKKESDESKSAFSFGSKPTGKEEGDGAKAAISFGAKPEEQKSSDTSKPAFTFGAQKDNEKKTEESSTGK
288	DP00814	MSDAAVDTSSEITTKDLKEKKEVVEEAENGREAPANGNANEENGEQEADNEVDEEEEEGGEEEEEEEEGDGEEEDGDEDEEAEAATGKRAAEDDEDDDVDTKKQKTDEDD
289	DP00256	MMPANEDKENNIVYTGNESSGINFPQTPAHLLKRSHSNILKPPVRLDQLKRDANSNNGNTLKYIQGGKEVSPTKRLHTHAQQQGRLPLAAKDNNRSKSFIFPETSNQSKD
290	DP00904	EKEIDEGALYIVSNKKLFKKLKNKNPNLKVVCTEGMLDIEDMRAIGVPEKALEGLKKKVEIARKNVERFIEKYKPEKIFVVVEDDKDELLYLRAKNLYNAEKLDADEILD
291	DP00184	FDRNVPRICGVCGDRATGFHFNAMTCEGCKGFFRRSMKRKALFTCPFNGDCRITKDNRRHCQACRLKRCVDIGMMKEFILTDEEVQRKREMILKRKEEEALKDSLRPKLS
292	DP00088	AVVPHVHERDAFWEMKEKGEAYKKPDHYEEIHMPKNSGAGIVIAAFSTIFGFAMIWHIWWLAIVGFAGMIITWIVKSFDEDVDYYVPVAEIEKLENQHFDEITKAGLKNGN
293	DP00706	GDSSGFSSYSGSSSSGSSISSARSSGGGSSGSSSGSSIAQGGSAGSFKPGTGYSQVSYSSGSGSSLQGASGSSQLGSSSSHSGNSGSHSGSSSSHSSSSSSFQFSSSSFQVG

294	DP00070	MDVFMKGLSKAKEGVVAAAEKTKQGVAEAAGKTKEGVLYVGSKTKEGVVHGVATVAEKTKEQVTNVGGAVVTGVTAVAQKTVEGAGSIAAATGFVKKDQLGKNEEGAPQEGIL
295	DP00518	MAKQPSDVSSECDREGGQLQPAERPPQLRPGAPTSLQTEPQGNPDGEGDRCPHGSPQGPLAPPASPGPFATRSPLFIFVRRSSLLSRSSSGYFSFDTDRSPAPMSCDKSTQTP
296	DP00621	CSSELYTEASGYISSLEYPRSYPPDLRCNYSIRVERGLTLHLKFLEPFDIDDHQQVHCPYDQLQIYANGKNIGEFCGKQRPPDLDTSSNAVDLLFFTDESGDSRGWKLRYTTE
297	DP00025	QNSGNQSFEEDTEEDKPKYEQGGNIVDIDFDSVPQIHGQNKGNQSFEEDTEKDKPKYEHGGNIIDIDFDSVPHIHGFNKHTEIIEEDTNKDKPSYQFGGHNSVDFEEDTLPKV
298	DP00066	MNRGFFNMLGRRPFPAPTAMWRPRRRRQAAPMPARNGLASQIQQLTTAVSALVIGQATRPQNPRPRPPPRQKKQAPKQPPKPKKPKPQEKKKKQPAKTKPGKRQRMALKLEAD
299	DP00200	RVKFSRSADAPAYQQGQNQLYNELNLGRREEYDVLDKRRGRDPEMGGKPQRRKNPQEGLYNELQKDKMAEAYSEIGMKGERRRGKGHDGLYQGLSTATKDTYDALHMQALPPR
300	DP00666	KRSKGGKYSVKDKEDTQVDSEARPMKDETFGEYRSLESDNEEKAFGSSQPSLNGDIKPLGSDDSLADYGGSVDVQFNEDGSFIGQYSGKKEKEAAGGNDSSGATSPINPAVALE
301	DP00531	MEEEKHHHHHLFHHKDKAEEGPVDYEKEIKHHKHLEQIGKLGTVAAGAYALHEKHEAKKDPEHAHKHKIEEEIAAAAAVGAGGFAFHEHHEKKDAKKEEKKKLRGDTTISSKLLF
302	DP00173	MEVTGDAGVPESGEIRTLKPCLLRRNYSREQHGVAASCLEDLRSKACDILAIDKSLTPVTLVLAEDGTIVDDDDYFLCLPSNTKFVALASNEKWAYNNSDGGTAWISQESFDVDET
303	DP00850	HHNKEWLSKPTDKRRLSELTQIWWDLKGKHEEHGKSNLGKIEIFTKITNDPCAFQITKSLISQYCATRRSQGIKPSSINRDLTCISGMFTALIEAELFFGEHPIRGTKRLKEEKPE
304	DP00625	DLEVIISLGPDPTRLDAKLLDSYSTAATSSSKDVISVATDVSNTITKTSDERLITEGEANQGVTATTVKATESDGNVPKAMTGSIDLDKEGIFDSVGITTIDPEVLKEKPWRQPGA
305	DP00387	MKKRNRLKKNEDFQKVFKHGTSVANRQFVLYTLDQPENDELRVGLSVSKKIGNAVMRNRIKRLIRQAFLEEKERLKEKDYIIIARKPASQLTYEETKKSLQHLFRKSSLYKKSSSK
306	DP00205	MKTTLIKVIAASVTALFLSMQVYASGHTAHVDEAVKHAEEAVAHGKEGHTDQLLEHAKESLTHAKAASEAGGNTHVGHGIKHLEDAIKHGEEGHVGVATKHAQEAIEHLRASEHKSH
307	DP00134	APTEEERESFLRRGDGRRRGGGGRGQGGRGRGGGFKGNDDHSRTDNRPRNPREAKGRTTDGSLQIRVDCNNERSVHTKTLQNTSSEGSRLRTGKDRNQKKEKPDSVDGQQPLVNGVP
308	DP00341	EDKPQDYKKRLSVELTSSLFRTEPMAWNETADLGLDIGAQGEALGYRQDDPSYRSFHSGGYGQDALGMDPMMEHEMGGHHPGADYPVDGLPDLGHAQDLMDGLPPGDSNQLAWFDTDL
309	DP01060	MGSLEMVPMGAGPPSPGGDPDGDDGGNNSQYPSASGSSGNTPTPPNDEERESNEEPPPPYEDPYWGNGDRHSDYQPLGTQDQSLYLGLQHDGNDGLPPPPYSPRDDSSQHIYEEAGRG
310	DP00553	YRKDKRRQEPLRQPSPQRGAGAPELGAAPEEELAALQLGPTHHECEAGPPHDTLRLTALPDYTLTLRRSPDDIPLMTPNTITMIPNSLVGLQTLHPYNTFAAGFNSTGLPHSHSTTRV
311	DP00447	MEKFAPEFHGEDANNRATKFLESIKGKFTSPKDPKKKDSIISVNSTDIEVTKESPITSNSTIINPTNETDDNAGNKPNYQRKPLVSFKEDPIPSDNPFSKLYKETIETFDNNEEESSY
312	DP01068	DLNTTLPIRQTASIFKQPVTKVTNHPSNKVKSDPQRMNEQPRQLFWEKRLQGLSASDVTEQIIKTMELPKGLQGVGPGSNDETLLSAVASALHTSSAPITGQVSAAVEKNPAVWLNTSQ
313	DP00537	MDRSLGWQGNSVPEDRTEAGIKRFLEDTTDDGELSKFVKDFSGNASCHPPEAKTWASRPQVPEPRPQAPDLYDDDLEFRPPSRPQSSDNQQYFCAPAPLSPSARPRSPWGKLDPYDSSE
314	DP00399	GFVVSDSNVKPDQTFADVLAISQRTTHNTVAVTDDGTPHGVLLGLVTQRDYPIDLTQTETKVSDMMTPFSKLVTAHQDTKLSEANKIIWEKKLNALPIIDDDQHLRYIVFRKDYDRSQVC
315	DP00049	HVKKPYYDPSRTEKRESKCPTPGCDGTGHVNGLYPHHRSLSGCPHKDRVPPEILAMHENVLKCPTPGCTGRGHVNSNRNSHRSLSGCPIAAAEKLAKAQEKHQSCDVSKSNQASDRVLRP
316	DP00544	MAKSIRSKHRRQMRNVKREHFAKKDLDRLKRLASKAQELDLDNVVTMKSAEEIKNKPSTSASDADKGMEVDNTKKVFKKKTQQNEDGHYPQWMNQRAVKKQKVKVAKLKTKKKIGKKIKW
317	DP00871	MDPNTVSSFQDILLRMSKMQLESSSEDLNGMITQFESLKLYRDSLGEAVMRMGDLHSLQNRNEKWREQLGQKFEEIRWLIEEVRHKLKITENSFEQITFMQALHLLLEVEQEIRTFSFQLI
318	DP00839	PMPGALGASGSSGHELSALGGEGGLQSLLGNMSHSQLMQLIGPAGLGGLGGLGALTGPGLASLLGSSGPPGSSSSSSSSRSQSAAVTPSSTTSSTRATPAPSAPAAASATSPSPAPSSGNGA
319	DP01103	AEAESLTSAQKAKAEEKKRRKMSKGLPDKSEEEKSTMAKKLEQKPKGEGIPTTAKLKIDEFESNVNEVKDPYPSADFPGDDEEDEPEIPLSPRPRPLAELQLKEKAVPIPEASSFFIFSPT
320	DP00020	MSSPDDFETAPAEYVDALDPSMVVVDSGSAAVTAPSDSAAEVKANQNEENTGATAAETSEKVDQTEVEKKDDDDTTEVGVTTTTPSIADTAATANIASTSGASVTEPTTDDTAADEKKEQVSG
321	DP00958	MKLPVAQYSAPDGVEKSFAPIRDDPRYMTTEGRTTGPSDHVLNAGQIDRDKPSEPERTKDGSQLTYLGQLRTQLTGLQDDINEFLTGRMELAKNKKKAGADEKRIQEEINQLLDGGDGDEDAV
322	DP00629	SGGDGAYHEPTGGGAIEVALDNADIDLETEAHADQDARGWGGESGERWARQVSGGHFVTLHGAERLEEETNDEDVSDIERRIAMRLAERRQEDSATHGDEGRNNGVDHDEDDDAAAVAGIGGI
323	DP00787	ATINNAGFESGFSNWNETDPAAISSDAYSGSKSLKIQGSPARVYQVVDIQPNTEYTLSAYVLGKGQIGVNDLNGLFKNQTFNVSSWTKVTKTFTSANTNSLQVFAKHYNNTSDVRFDNFSLIEG
324	DP00012	IRKFSIVQKTPLQMNGIEEDSDEPLERRLSLVPDSEQGEAILPRISVISTGPTLQARRRQSVLNLMTHSVNQGQNIHRKTTASTRKVSLAPQANLTELDIYSRRLSQETGLEISEEINEEDLKE
325	DP00662	LTNNVLSTAQQVYLRKLGGAKPNMDENASKIISAGRAKRSIAQPDDAGERFRQLKEQEKRSKKNKAVAKDTVELVEESQSESEEGSDDEEEEAREGALASSTTSKPLPEVGQRRSKRSKRKRTV
326	DP00160	HTTEDKISRAVGPRQAQVSFLQGDQSENELPRLGGKEDRRVKQSRGEARESYRETGPSRASDARAAHLPTGTPLDIDTASESSQDPQDSRRSAEPLLSCKPWQESRKNKAQTRTPLQCTMTEIF
327	DP00629	LSGGDGAYHEPTGGGAIEVALDNADIDLETEAHADQDARGWGGESGERWARQVSGGHFVTLHGAERLEEETNDEDVSDIERRIAMRLAERRQEDSATHGDEGRNNGVDHDEDDDAAAVAGIGGI
328	DP00774	QTTDNVDELFDTQLMLFAKRLSTLDLNEINAADRMAQTPNRLKHGHVDDDALTFAIFTHDGRMVLNDGDNGEDIPYSYQREGFADGQLVGEDDPWRFVWMTSPDGKYRIVVGQEWEYREDMALA

329	DP00085	PTQAGEGTLSEALLQLQFDDEDLGALLGNSTDPAVFTDLASVDNSEFQQLLNQGIPVAPHTTEPMLMEYPEAITRLVTGAQRPPDPAPAPLGAPGLPNGLLSGDEDFSSIADMDFSALLSQISS
330	DP00418	RKRRKPGSHTHSASEDNTTNNVREQLNQIKNPIEKHGANTVPIKDYENKNSKMSKIRTHNSEVEEDDMDKHQQKARFAKQPAYTLVDREEKPPNGTPTKHPNWTNKQDNRDLESAQSLNRMEYIV
331	DP01100	QTAPVPMPDLKNVKSKIGSTENLKHQPGGGKVQIINKKLDLSNVQSKCGSKDNIKHVPGGGSVQIVYKPVDLSKVTSKCGSLGNIHHKPGGGQVEVKSEKLDFKDRVQSKIGSLDNITHVPGGGNK
332	DP00219	MAEAGAGLSETVTETTVTVTTEPENRSLTIKLRKRKPEKKVEWTSDTVDNEHMGRRSSKCCCIYEKPRAFGESSTESDEEEEEGCGHTHCVRGHRKGRRRATLGPTPTTPPQPPDPSQPPPGPMQH
333	DP01103	EAESLTSAQKAKAEEKKRRKMSKGLPDKSEEEKSTMAKKLEQKPKGEGIPTTAKLKIDEFESNVNEVKDPYPSADFPGDDEEDEPEIPLSPRPRPLAELQLKEKAVPIPEASSFFIFSPTNKIRVL
334	DP00630	MDVFKKGFSIAKEGVVGAVEKTKQGVTEAAEKTKEGVMYVGAKTKENVVQSVTSVAEKTKEQANAVSEAVVSSVNTVATKTVEEAENIAVTSGVVRKEDLRPSAPQQEGEASKEKEEVAEEAQSGGD
335	DP00546	MRRRGEIDMATEGDVELELETETSGPERPPEKPRKHDSGAADLERVTDYAEEKEIQSSNLETAMSVIGDRRSREQKAKQEREKELAKVTIKKEDLELIMTEMEISRAAAERSLREHMGNVVEALIALTN
336	DP01100	QTAPVPMPDLKNVKSKIGSTENLKHQPGGGKVQIINKKLDLSNVQSKCGSKDNIKHVPGGGSVQIVYKPVDLSKVTSKCGSLGNIHHKPGGGQVEVKSEKLDFKDRVQSKIGSLDNITHVPGGGNKKIE
337	DP00365	PPADGALKRAEELKTQANDYFKAKDYENAIKFYSQAIELNPSNAIYYGNRSLAYLRTECYGYALGDATRAIELDKKYIKGYYRRAASNMALGKFRAALRDYETVVKVKPHDKDAKMKYQECNKIVKQKA
338	DP00025	GQNSGNQSFEEDTEEDKPKYEQGGNIVDIDFDSVPQIHGQNKGNQSFEEDTEKDKPKYEHGGNIIDIDFDSVPHIHGFNKHTEIIEEDTNKDKPSYQFGGHNSVDFEEDTLPKVSGQNEGQQTIEEDTTP
339	DP00062	MDTKHFLPLDFSTQVNSSLTSPTGRGSMAAPSLHPSLGPGIGSPGQLHSPISTLSSPINGMGPPFSVISSPMGPHSMSVPTTPTLGFSTGSPQLSSPMNPVSSSEDIKPPLGLNGVLKVPAHPSGNMASF
340	DP00224	MHHQQRMAALGTDKELSDLLDFSAMFSPPVSSGKNGPTSLASGHFTGSNVEDRSSSGSWGNGGHPSPSRNYGDGTPYDHMTSRDLGSHDNLSPPFVNSRIQSKTERGSYSSYGRESNLQGCHQQSLLGGD
341	DP00534	MATIATGLNIATQRVFVTSENRPVCLAGPVHLNNSWNLGSRTTNRMMKLQPIKAAPEGGISDVVEKSIKEAQETCAGDPVSGECVAAWDEVEELSAAASHARDKKKADGSDPLEEYCKDNPETNECRTYDN
342	DP01104	RKRRRQHGQLWFPEGFKVSEASKKKRREPLGEDSVGLKPLKNASDGALMDDNQNEWGDEDLETKKFRFEEPVVLPDLDDQTDHRQWTQQHLDAADLRMSAMAPTPPQGEVDADCMDVNVRGPDGFTPLMIA
343	DP00341	MATQADLMELDMAMEPDRKAAVSHWQQQSYLDSGIHSGATTTAPSLSGKGNPEEEDVDTSQVLYEWEQGFSQSFTQEQVADIDGQYAMTRAQRVRAAMFPETLDEGMQIPSTQFDAAHPTNVQRLAEPSQMLK
344	DP00698	SVGRQDNNMQAREAKFAAGGVLVGGGEQDIDEEEEPIEHSGRQSVTFKREMSMSSLADSVPSSSVSTSGGTRLTNSLLNLRSRLAAKAIKESTAQSSSERNPPNNRPQADSGRKDDQEPKPAQNDLDFVRADV
345	DP00555	MDVFMKGLSMAKEGVVAAAEKTKQGVTEAAEKTKEGVLYVGSKTREGVVQGVASVAEKTKEQASHLGGAVFSGAGNIAAATGLVKREEFPTDLKPEEVAQEAAEEPLIEPLMEPEGESYEDPPQEEYQEYEPEA
346	DP00122	MADERKDEGKAPHWTSASLTEAAAHPHSPEMKDQGGSGEGLSRSANGFPYREEEEGAFGEHGSQGTYSDTKENGINGELTSADRETAEEVSARIVQVVTAEAVAVLKGEQEKEAQHKDQPAALPLAAEETVNLPP
347	DP00120	RLEQYTSAVVGNKAAKPAKPAASDLPVPAEGVRNIKSMWEKGNVFSSPGGTGTPNKETAGLKVGVSSRINEWLTKTPEGNKSPAPKPSDLRPGDVSGKRNLWEKQSVEKPAASSSKVTATGKKSETNGLRQFEKEP
348	DP00965	KKDKDGEGAPPAKRARTDQMEVDSGPRKRPSRGGFTDKERQDHRRRKALENKRKQLSAGGKNLSKEEEEELRRLTEEDERRERRIAGPQVGGVNPLEGGTRGAPGGGFVPSMQGVPESPFTRTGEGLDIRGSQGFP
349	DP00340	MPNFAGTWKMRSSENFDELLKALGVNAMLRKVAVAAASKPHVEIRQDGDQFYIKTSTTVRTTEINFKVGEGFEEETVDGRKCRSLPTWENENKIHCTQTLLEGDGPKTYWTRELANDELILTFGADDVVCTRIYVRE
350	DP00794	SSVSSSPSPPFGHSAAVSPTFMPRSTQPLTFTTATFAATKFGSTKMKNSGRSNKVARTSPINLGLNVNDLLKQKAISSSMHSLYGLGLGSQQQPQQQQQPAQPPPPPPPPQQQQQQKTSALSPNAKEFIFPNMQGQGSS
351	DP00626	MLFIKPADLREIVTFPLFSDLVQCGFPSPAADYVEQRIDLNQLLIQHPSATYFVKASGDSMIDGGISDGDLLIVDSAITASHGDIVIAAVDGEFTVKKLQLRPTVQLIPMNSAYSPITISSEDTLDVFGVVIHVVKAMR
352	DP00070	MDVFMKGLSKAKEGVVAAAEKTKQGVAEAAGKTKEGVLYVGSKTKEGVVHGVATVAEKTKEQVTNVGGAVVTGVTAVAQKTVEGAGSIAAATGFVKKDQLGKNEEGAPQEGILEDMPVDPDNEAYEMPSEEGYQDYEPEA
353	DP00196	AVPVESKPDKPSGKSGMDAALDDLIDTLGGPEETEEENTTYTGPEVSDPMSSTYIEELGKREVTIPPKYRELLAKKEGITGPPADSSKPIGPDDAIDALSSDFTCGSPTAAGKKTEKEESTEVLKAQSAGTVRSAAPPQEK
354	DP00999	CIFEVKHEGKVTGYACLVGDKVMKPAHVKGVIDNADLAKLAFKKSSKYDLECAQIPVHMRSDASKYTHEKPEGHYNWHHGAVQYSGGRFTIPTGAGKPGDSGRPIFDNKGRVVAIVLGGANEGSRTALSVVTWNKDMVTRVT
355	DP00134	FAEDVIQVPRNLVGKVIGKNGKLIQEIVDKSGVVRVRIEAENEKNVPQEEEIMPPNSLPSNNSRVGPNAPEEKKHLDIKENSTHFSQPNSTKVQRVLVASSVVAGESQKPELKAWQGMVPFVFVGTKDSIANATVLLDYHLN
356	DP00186	MSSQQNQNRQGEQQEQGYMEAAKEKVVNAWESTKETLSSTAQAAAEKTAEFRDSAGETIRDLTGQAQEKGQEFKERAGEKAEETKQRAGEKMDETKQRAGEMRENAGQKMEEYKQQGKGKAEELRDTAAEKLHQAGEKVKGRD
357	DP00260	MPLNVSFTNRNYDLDYDSVQPYFYCDEEENFYQQQQQSELQPPAPSEDIWKKFELLPTPPLSPSRRSGLCSPSYVAVTPFSLRGDNDGGGGSFSTADQLEMVTELLGGDMVNQSFICDPDDETFIKNIIIQDCMWSGFSAAAK
358	DP00378	TTSGSSGTNSQGQTPQRVSGLQGSDALNIQQNQTSGGSLQAGQQKEGEQNQQTQQQQILIQPQLVQGGQALQALQAAPLSGQTFTTQAISQETLQNLQLQAVPNSGPIIIRTPTVGPNGQVSWQTLQLQNLQVQNPQAQTITLAPMQ
359	DP00378	TNSQGQTPQRVSGLQGSDALNIQQNQTSGGSLQAGQQKEGEQNQQTQQQQILIQPQLVQGGQALQALQAAPLSGQTFTTQAISQETLQNLQLQAVPNSGPIIIRTPTVGPNGQVSWQTLQLQNLQVQNPQAQTITLAPMQGVSLGQT
360	DP00092	SDDELGSEEDGFDGATAAARKEVIRNKIRAIGKMARVFSVLREESESVLTLKGLTPTGMLPSGVLSGGKQTLQSATVEAIEADEAIKGFSPQHKITSFEEAKGLDRINERMPPRRDAMPSDANLNSINKALTSETNGTDSNGSNSSNIQ
361	DP00174	MASSDIQVKELEKRASGQAFELILSPRSKESVPEFPLSPPKKKDLSLEEIQKKLEAAEERRKSHEAEVLKQLAEKREHEKEVLQKAIEENNNFSKMAEEKLTHKMEANKENREAQMAAKLERLREKDKHIEEVRKNKESKDPADETEAD
