# Supplementary material for: Sequence Versus Composition: What Prescribes IDP Biophysical Properties?
Source: Entropy (Basel). 2019 Jul 3;21(7):654. doi: 10.3390/e21070654 (PMC7515148; doi:10.3390/e21070654)
Supplement: Supplementary file 1 [file entropy-21-00654-s001.zip › SuplMaterial/Vymetal_TableS2.pdf]

Table S2: Justification of secondary structure predictors measured on PDB dataset (the reported numbers represent fraction of correctly predicted residues in a three state model - helix, sheet, coil)

method	consensus	spider3	psipred	predator	jnet	simpa	GOR IV
precision[%]	73.0	78.0	72.0	65.8	65.1	68.4	63.2
