# Supplementary figures and images for: Sequence Versus Composition: What Prescribes IDP Biophysical Properties?
Source: Entropy (Basel). 2019 Jul 3;21(7):654. doi: 10.3390/e21070654 (PMC7515148; doi:10.3390/e21070654)

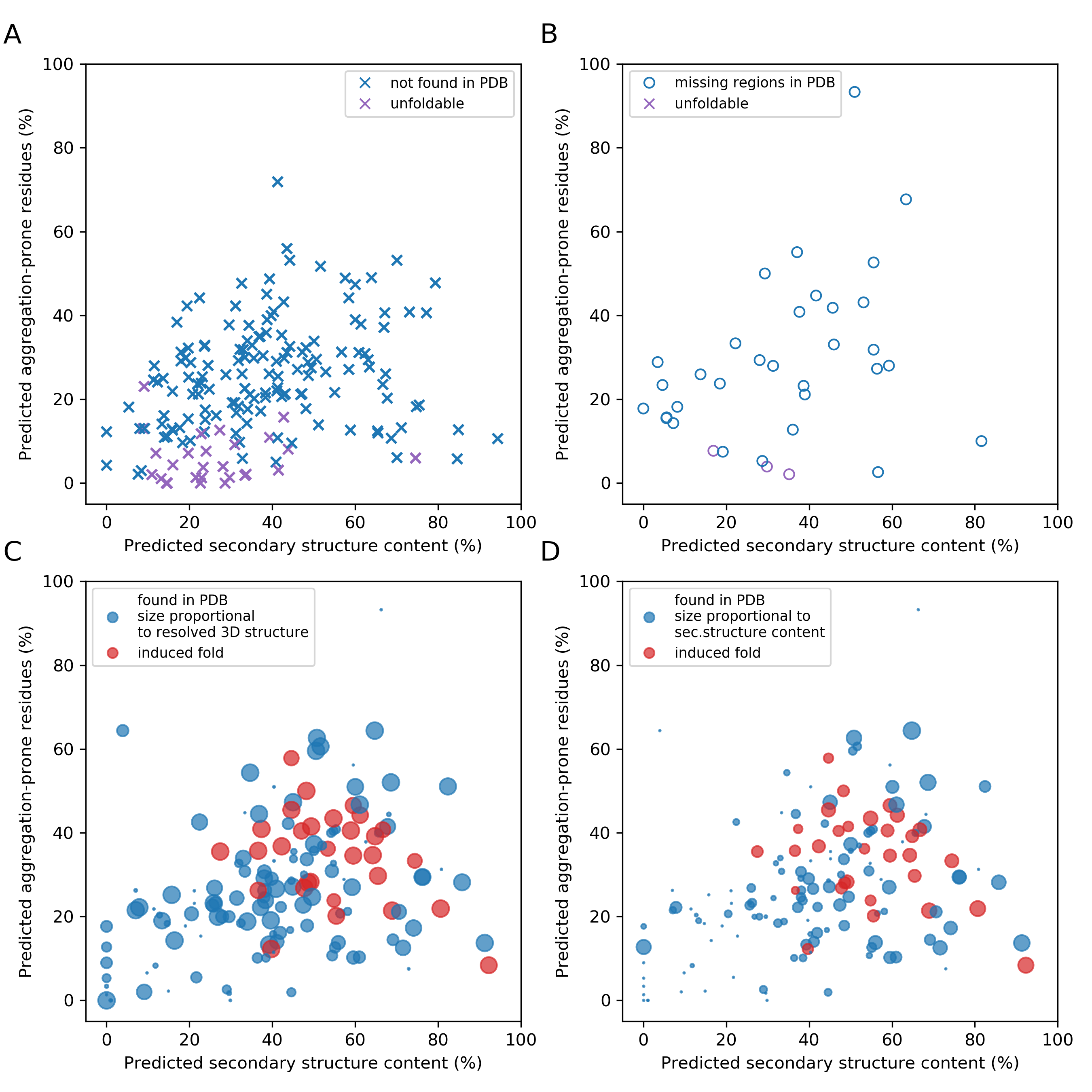

Supplement: Supplementary file 1 [file entropy-21-00654-s001.zip › SuplMaterial/figureS1.png]

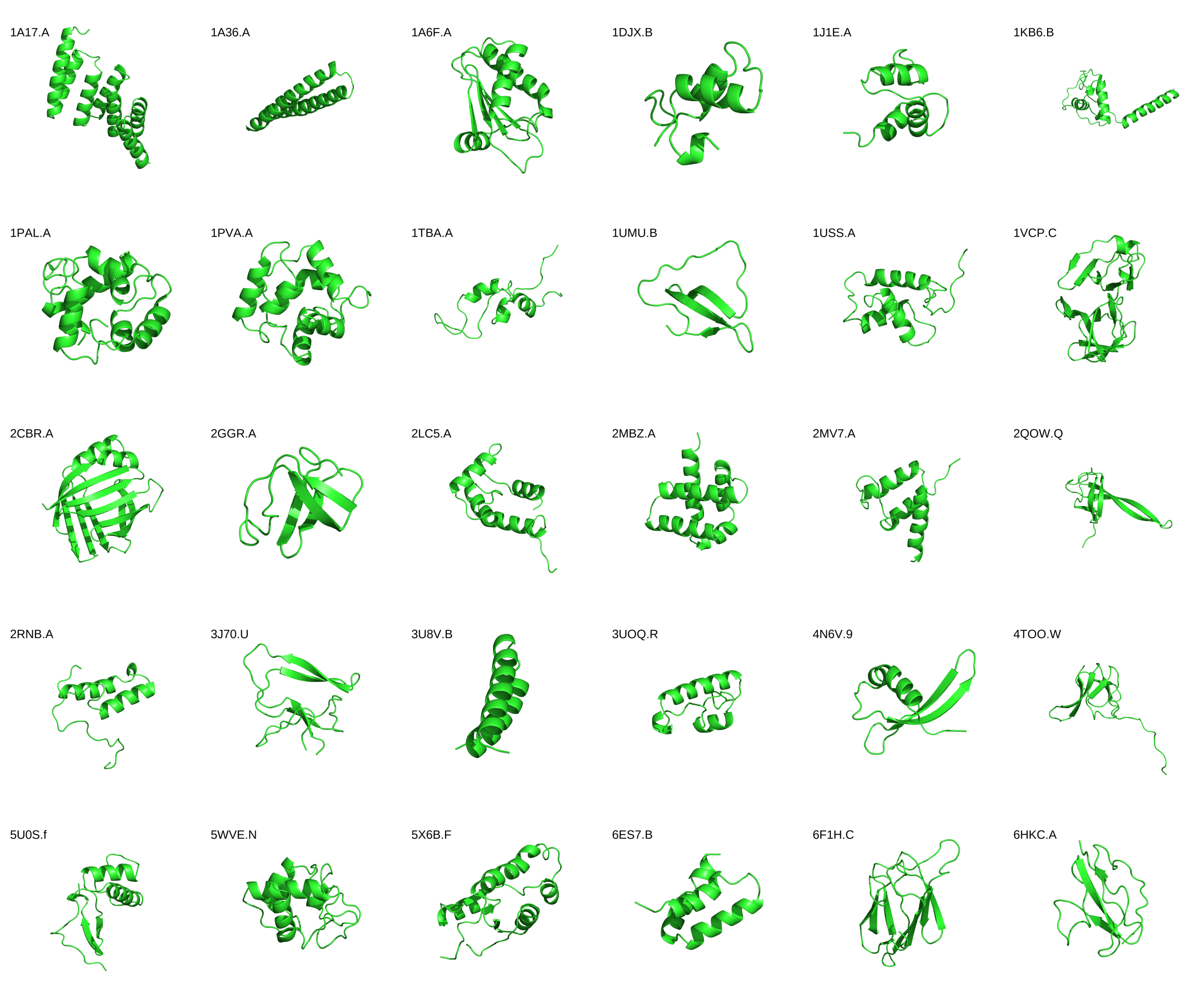

Supplement: Supplementary file 1 [file entropy-21-00654-s001.zip › SuplMaterial/figureS2.png]

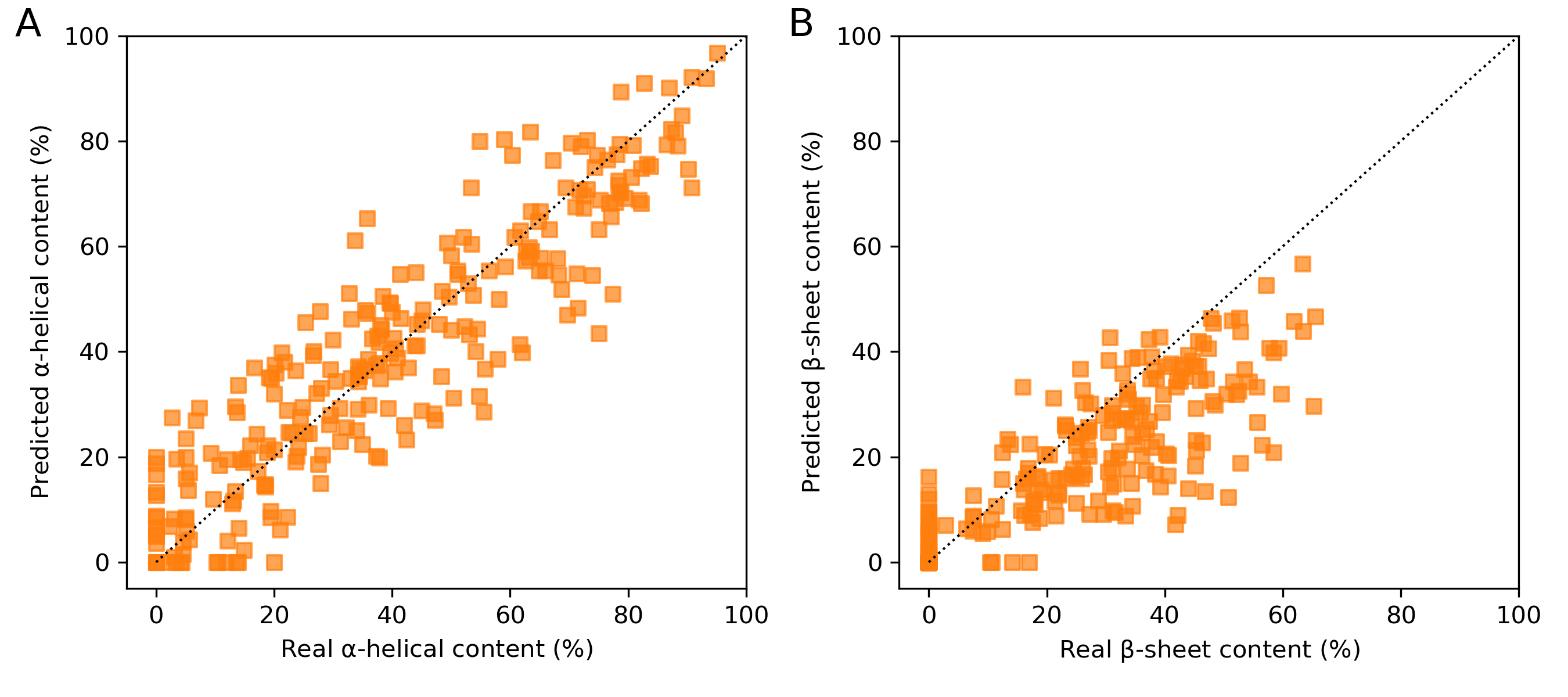

Supplement: Supplementary file 1 [file entropy-21-00654-s001.zip › SuplMaterial/figureS3.png]
